# High‐Valence Oxides for High Performance Oxygen Evolution Electrocatalysis

**DOI:** 10.1002/advs.202301706

**Published:** 2023-05-30

**Authors:** Hao Wang, Tingting Zhai, Yifan Wu, Tao Zhou, Binbin Zhou, Congxiao Shang, Zhengxiao Guo

**Affiliations:** ^1^ Department of Chemistry The University of Hong Kong Hong Kong SAR 000000 China; ^2^ Green Catalysis Center College of Chemistry Zhengzhou University Zhengzhou 450001 China; ^3^ Department of Mechanical Engineering The University of Hong Kong Hong Kong SAR 000000 China; ^4^ Shenzhen Institute of Advanced Electronic Materials Shenzhen Institute of Advanced Technology Chinese Academy of Sciences Shenzhen 518055 China; ^5^ Zhejiang Institute of Research and Innovation The University of Hong Kong Hangzhou 311300 China

**Keywords:** oxygen evolution reaction, high‐valence oxides, valence tuning, electrocatalysis

## Abstract

Valence tuning of transition metal oxides is an effective approach to design high‐performance catalysts, particularly for the oxygen evolution reaction (OER) that underpins solar/electric water splitting and metal‐air batteries. Recently, high‐valence oxides (HVOs) are reported to show superior OER performance, in association with the fundamental dynamics of charge transfer and the evolution of the intermediates. Particularly considered are the adsorbate evolution mechanism (AEM) and the lattice oxygen‐mediated mechanism (LOM). High‐valence states enhance the OER performance mainly by optimizing the *e*_g_‐orbital filling, promoting the charge transfer between the metal d band and oxygen p band. Moreover, HVOs usually show an elevated O 2p band, which triggers the lattice oxygen as the redox center and enacts the efficient LOM pathway to break the “scaling” limitation of AEM. In addition, oxygen vacancies, induced by the overall charge‐neutrality, also promote the direct oxygen coupling in LOM. However, the synthesis of HVOs suffers from relatively large thermodynamic barrier, which makes their preparation difficult. Hence, the synthesis strategies of the HVOs are discussed to guide further design of the HVO electrocatalysts. Finally, further challenges and perspectives are outlined for potential applications in energy conversion and storage.

## Graphical abstract

High valence oxides of transition metals represent an emerging group of valence‐engineered catalysts, capable of offering very high catalytic activity and stability. The mechanisms of such “unconventional” characteristics and their synthesis strategies are discussed here. The case for oxygen evolution reaction is reviewed to guide effective development of the catalytic structures for water‐splitting hydrogen generation and metal‐air batteries.

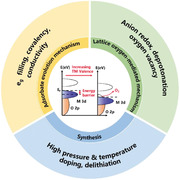

## 1 Introduction

Oxygen evolution reaction (OER) is a critical step in water electrolysis for clean hydrogen generation and in rechargeable metal‐air batteries.^[^ ^1^, ^2^ ^]^ Both are important for enhancing the electrification of transport, the interconnectedness of things, and the efficiency and the integration of renewable energy sources to the electric grid, ultimately to reduce harmful emissions from the burning of fossil fuels while ensuring security of energy supply for sustainable economic and societal development. Due to multi‐step 4‐electron transfer pathways, OER usually suffers from a relatively slow reaction kinetics and a high overpotential, unfavorable for practical applications.^[^ ^3^ ^]^ Noble‐metal oxides, IrO_2_ and RuO_2_, are the benchmark for catalyzing the OER process, often occupying the apex position of the OER activity volcano plot among most oxides. However, the high cost of the noble elements and unsatisfactory stability of the oxides also restrict their applications.^[^ ^4^, ^5^ ^]^ Transition metal oxides (TMOs), e.g., based on Ni, Fe, and Co, have emerged as promising alternatives due to their earth‐abundance and thus low cost.^[^ ^6^ ^]^ Despite considerable processes, their high overpotential and low stability are major issues to be addressed before commercial applications. It is recently demonstrated that valence state engineering is an effective strategy to enhance the intrinsic OER activity and stability of TMOs. Some HVOs are reported to show superior OER activity than their low‐valence counterparts.^[^ ^7^, ^8^ ^]^ For instance, the Fe^4+^‐based quadruple perovskite CaCu_3_Fe_4_O_12_ shows higher OER activity than LaFeO_3_ with Fe^3+^.^[^ ^9^ ^]^ CaCoO_3_, with Co^4+^, exhibits better OER activity than the Co^3+^‐based oxides, such as LaCoO_3_ and Co_3_O_4_.^[^ ^10^ ^]^ Hg_2_Ru_2_O_7_, with Ru^5+^, displays much better OER activity than the commercial RuO_2_, with an ultra‐small overpotential of 150 mV at 10 mA cm^−2^.^[^ ^11^ ^]^

However, the exact mechanism of the high‐valence effect has not been fully established, nor the catalysis pathways. Based on the traditional adsorbate evolution mechanism (AEM), the d‐band center theory offers some general guidance to the catalyst design,^[^ ^12^ ^]^ and more recently, the oxygen p‐band center is also proposed as an alternative descriptor for OER,^[^ ^13^ ^]^ e.g., promoting TMOs’ O p‐band close to the Fermi level creates highly active OER catalysts from double‐perovskites.^[^ ^13^ ^]^ The O p‐band relative to the Fermi level reflects the extend of its overlap with the M d‐band in the M—O bond, which directly influences the electron transfer therein. Further analysis indicates that for a given type of TMOs, a higher valence leads to a stronger covalency of the M–O interaction, which typically induces a downshift of the metal d‐band and/or promotion of the O p‐band, to create a larger overlap of the two, which facilitates the charge transfer and hence ready removal of protons in the first step of the OER process.^[^ ^14^ ^]^ The electronic configuration, especially the *e*_g_ orbital filling, significantly influences the binding strength of oxygen intermediates, and further influences the intrinsic OER activities. Shao–Horn's group has proposed two descriptors for perovskite oxides: *e*_g_ filling of the B‐site metal and the covalency of the B—O or M—O bond^25^ (M for the metal atom at the B site). The optimized *e*_g_ filling for OER is close to unity because a lower or higher *e*_g_ filling (*e*_g_ < 1, or *e*_g_ > 1) results in too strong or too weak a binding with oxygen; neither favors the OER process. Furthermore, the electrical conductivity determines the electron transport between the catalyst and the current collector, which influences the overall OER catalytic kinetics.

Although the descriptors via the AEM pathway have been well investigated, the AEM suffers from a strong “scaling limitation” with a minimum overpotential of 0.37 ± 0.1 V due to the linear correlation of the adsorbates OOH* and OH*,^[^ ^15^ ^]^ involving a given active site and the same “M—*O” bond coordination. In order to bypass this limitation, this “single‐site/single‐bond coordination” must be de‐coupled for the OER process. Several new OER mechanisms have been strategized to break the constraint. The most developed is the so‐called lattice oxygen‐mediated mechanism (LOM), where a lattice oxygen ligand is activated and serves as part of a dual‐site M‐O redox center to donate electrons directly to the external circuit with the holes retained in the oxygen 2p band. This M–O dual‐site facilitates the O–O coupling to generate the O_2_^−^ species instead of the *OOH via the AEM pathway. The LOM mechanism effectively breaks the “single‐site” coordination, and enact a “M+O” dual site reaction pathway without the need for the *OOH generation at the same M site, so that the “scaling” limitation is broken and the OER pathway can be further tuned to enhance the intrinsic activity. It is demonstrated that a strong covalency of the M—O bond, facilitated by a high concentration of oxygen vacancies, energetically promote this efficient OER mechanism. HVOs possess a higher state hybridization of the metal d band and the oxygen p band, which upshifts the O 2p band toward the Fermi level and triggers the lattice oxygen as the active center. Moreover, due to the existence of the non‐concerted proton electron transfer step via LOM, the deprotonation property should be also considered. A rapid deprotonation step is desirable for LOM to break the traditional concerted electron‐proton transfer step in AEM. Metal sites with higher oxidation state possess higher electrophilicity and stronger attraction for the O‐2p electrons, which will weaken the O—H bond and make proton dissociation more readily. Hence, HVOs facilitate the strong covalency, large concentration of oxygen vacancies and ready deprotonation, to catalyze oxygen evolution via an efficient LOM pathway.

Generally speaking, simple stable metal oxides are in relatively low‐valence states. Extra energy input, e.g., via electric potential (voltage), high temperature or high pressure, is required to elevate the valence states of cations due to their relatively high chemical potential. Hence, TMOs with high valence states suffer from a relatively large thermodynamic formation barrier and usually not stable at ambient conditions.^[^ ^16^, ^17^ ^]^ However, some HVOs have been identified to be kinetically stable and can retain their structures after the removal of the applied energy, such as Hg_2_Ru_2_O_7_, CaCu_3_Fe_4_O_12_, and CaCoO_3_.^[^ ^18^ ^]^ Considerable effort has been devoted to overcoming the large formation barriers for synthesis and a few strategies have been proposed to develop high valence oxides with stability. For instance, the valence of transition metal in the perovskite oxides (ABO_3_) have been engineered via chemical doping by low‐valence or high‐electronegativity cations to obtain a mixed‐valence B^3+/4+^ compound (such as Sr_x_La_1−x_CoO_3_,^[^ ^19^ ^]^ Sr_x_La_1−x_Fe_y_Co_1−y_O_3_^[^ ^20^ ^]^), which can significantly enhance their OER performance. Moreover, the catalytic activity of LiCoO_2_ is significantly improved with the rise of the Co oxidation state, which can be achieved by the reduction of the Li content via electrochemical^[^ ^21^ ^]^ or chemical^[^ ^22^ ^]^ de‐lithiation to generate Li_0.5_CoO_2_ with the Co^3+/4+^. In another report, the synthesis in a diamond anvil under high temperature and high pressure is reported for high‐valence TMOs, such as CaCu_3_Fe_4_O_12_ (Fe^4+^),^[^ ^9^ ^]^ Hg_2_Ru_2_O_7_ (Ru^5+^),^[^ ^11^ ^]^ and CaCoO_3_ (Co^4+^),^[^ ^23^ ^]^ all of which exhibit improved OER performance, over the corresponding low‐valence oxides. The main drawback of this method is that only a small quantity of HVOs, usually milligrams, are produced under extreme experimental conditions, which is impractical to scale up.

**Figure 1:**
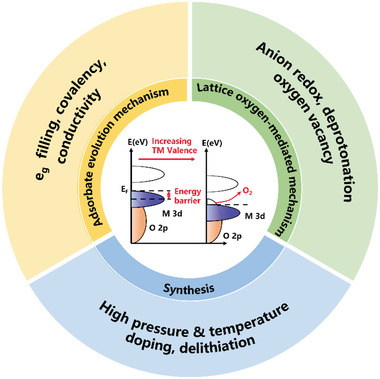
Design and synthesis strategies of high‐valence oxides as OER electrocatalysts.

It is of great significance to understand the fundamental reaction mechanisms and formulate strategies to synthesize HVOs under mild conditions with high yield, in order to realize the full potential and benefit of such catalysts. Here, we summarized the recent progresses in HVOs as OER catalysts from fundamental and experimental considerations (**Figure** **1**). After a brief introduction of the OER pathway and some important criteria to evaluate OER performance, the crucial roles of high‐valence transition metal sites in OER were discussed, including the optimization of the electronic band structures to reduce the energy barrier to promote the charge transfer along the AEM pathway, and the triggering of a LOM pathway to break the “scaling” limitation. Then the strategies to develop HVOs were carefully reviewed, including chemical doping, high‐pressure synthesis in the diamond anvil, and de‐lithiation/de‐sodiation of layered oxides, such as LiCoO_2_ and NaFe_x_Ni_1−x_O_2_. Then the advances of the HVOs with different crystalline structures were discussed. Finally, we highlighted the remaining challenges and future prospect for practice applications of high‐valence TMOs.

## 2 Brief Introduction of OER

### 2.1 Reaction Mechanisms

**Figure 2:**
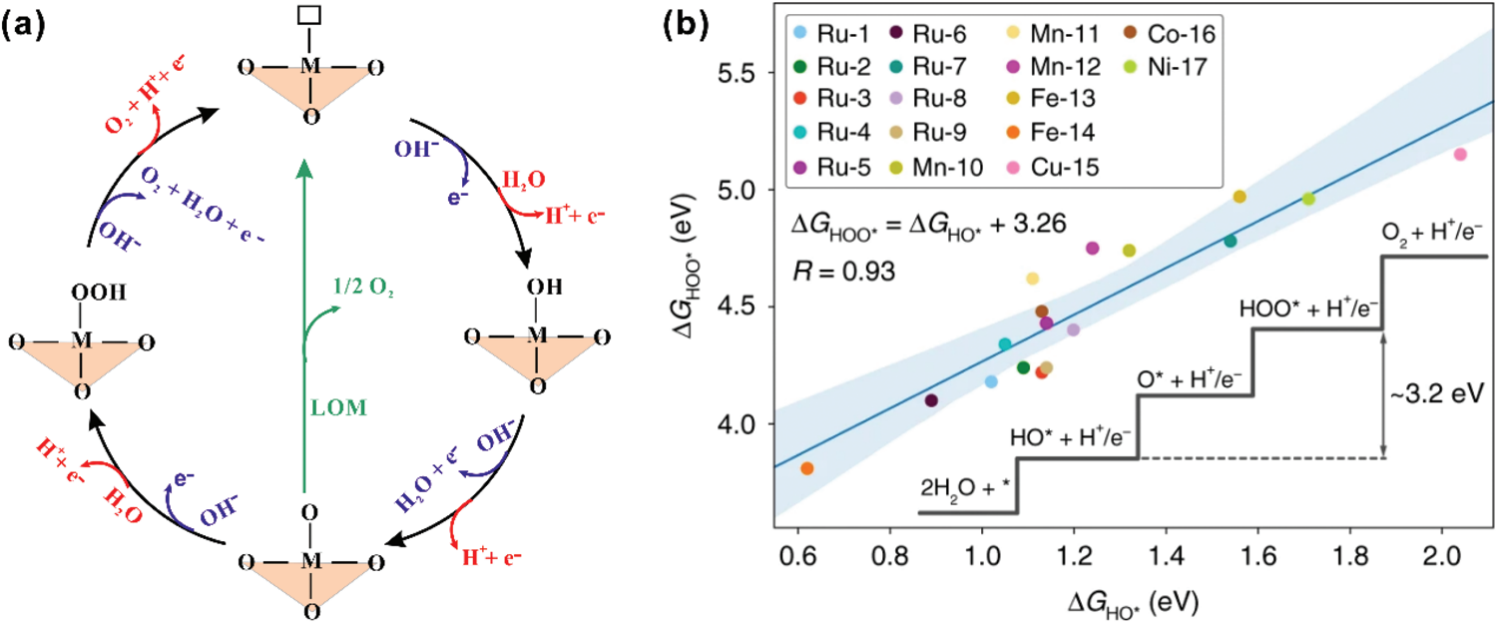
a) Schematic of the OER pathways for oxides in acidic (red route) and alkaline (blue route) conditions. The black line indicates the adsorbates evolution mechanism (AEM), while the green line indicates lattice oxygen‐mediated mechanism (LOM). b) Example of the linear scaling relation of binding energies between the HO∗ and HOO∗ intermediates for molecular OER catalysts. Reproduced under the terms of the Creative Commons Attribution 4.0 International License.^[^ ^26^ ^]^ Copyright 2019, The Authors, published by Springer Nature.

The adsorbate evolution mechanism (AEM) is widely accepted as the conventional OER pathway, where four concerted proton–electron transfer (CPET) reactions with three adsorbed intermediates: OH*, O*, and OOH*, are involved in either acidic or alkaline conditions (**Figure** **2**a).^[^ ^24^ ^]^

The overall reaction in an acidic solution:

(1) $$2H_{2}O\to O_{2}+4H^{+}+4e^{-}$$

with the four‐electron transfer steps:

(2) $$2H_{2}O\to {\mathrm{OH}}^{*}+H^{+}+e^{-}+H_{2}O$$

(3) $${\mathrm{OH}}^{*}+H^{+}+e^{-}+H_{2}O\to O^{*}+2H^{+}+2e^{-}+H_{2}O$$

(4) $$O^{*}+2H^{+}+2e^{-}+H_{2}O\to {\mathrm{OOH}}^{*}+3H^{+}+3e^{-}$$

(5) $${\mathrm{OOH}}^{*}+3H^{+}+3e^{-}\to O_{2}+4H^{+}+4e^{-}$$

For reaction in an alkaline solution:

(6) $$4{\mathrm{OH}}^{-}\to O_{2}+2H_{2}O+4e^{-}$$

with the corresponding four‐electron transfer steps:

(7) $$4{\mathrm{OH}}^{-}\to {\mathrm{OH}}^{*}+3{\mathrm{OH}}^{-}+e^{-}$$

(8) $${\mathrm{OH}}^{*}+3{\mathrm{OH}}^{-}\to O^{*}+2{\mathrm{OH}}^{-}+H_{2}O+e^{-}$$

(9) $$O^{*}+2{\mathrm{OH}}^{-}+H_{2}O\to {\mathrm{OOH}}^{*}+{\mathrm{OH}}^{-}+H_{2}O+e^{-}$$

(10) $${\mathrm{OOH}}^{*}+{\mathrm{OH}}^{-}+H_{2}O\to O_{2}+2H_{2}O+e^{-}$$

In both cases, each step is accompanied by the deprotonation and electron transfer to the external circuit. The hydroxy from H_2_O (acidic solution) or OH^−^ (alkaline solution) is firstly adsorbed on a metal active site on the surface of a catalyst (Equations (2) and (7)). The adsorbed OH* will go through deprotonation to form another intermediate O* with the transfer of an electron (Equations (3) and (8)). The metal d band is regarded as the redox center to donate electrons to the external circuit, and then the electrons from O 2p band is transferred to refill the holes in the metal d band.^[^ ^13^, ^14^ ^]^ Hence, a large overlap between the metal d band and O 2p band will facilitate the electron transfer in this step. Then the O* will be attacked by another hydroxy in the electrolyte to form the intermediate OOH* (Equations (4) and (9)). In the final step, OOH* is deprotonated to release the oxygen molecule (Equations (5) and (10)), with the active site re‐generated for the next OER cycle. As a series of adsorption and desorption steps of the oxygen intermediates are involved, an ideal OER catalyst should bind each of the oxygen species “neither too strongly nor too weakly,” as depicted by the Sabatier principle.^[^ ^25^ ^]^

Many OER catalysts have been designed and optimized, based on the AEM pathway. Several OER descriptors have been proposed, among which the *e*_g_‐orbital filling (*e*_g_‐filling) and the covalency of the metal (M)—O bond are widely accepted. However, AEM suffers from a “scaling relationship” with a minimum overpotential of 0.37 ± 0.1 V because of the linear correlation of the binding energies of the adsorbates OOH* and OH*^[^ ^15^ ^]^ at a given active site (Figure 2b).^[^ ^26^ ^]^ Recently, several new OER mechanisms have been proposed to bypass this scaling limitation. Of the most importance is the lattice oxygen‐mediated mechanism (LOM), where a lattice oxygen (vacancy site) is triggered and acts as part of the redox center to donate electrons to the external circuit, with the holes left in the oxygen 2p band, which further facilitates the O–O coupling to generate the intermediate *OO, instead of the *OOH via the AEM pathway. As such, the LOM breaks the “scaling” correlation of AEM due to the transfer from a single M active site to a dual M–O active site that avoided the *OOH and achieve a better intrinsic activity. Hence, catalysts with a strong M—O bond covalency and large concentration of O vacancies are reported to be more energetically favorable for LOM. Currently, it is unclear which of these two mechanisms may dominate experimentally for a given system, and the OER catalysis may proceed proportionately with these two pathways depending on the catalyst.

### 2.2 Brief Comments on Performance Indicators for OER

**Figure 3:**
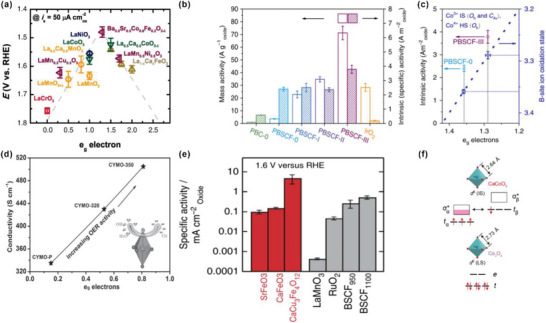
a) OER catalytic activity, defined by the overpotential at 50 µA cm^−2^_ox_, and the occupancy of the *e*_g_‐symmetry electron of the transition metal (B in ABO_3_). Reproduced with permission.^[^ ^14^ ^]^ Copyright 2011, American Association for the Advancement of Science. b) Mass activities and BET surface area‐normalized intrinsic activities of catalysts at *η* = 0.37 V derived from activity curves. c) Intrinsic activity versus *e*_g_ electron filling of PBSCF‐0, III. Reproduced under the terms of the Creative Commons Attribution 4.0 International License.^[^ ^42^ ^]^ Copyright 2017, The Authors, published by Springer Nature. d) The OER activity increased with conductivity and *e*_g_ electrons filling status optimized by hydrogen treatment. Reproduced with permission.^[^ ^43^ ^]^ Copyright 2015, Wiley‐VCH. Inset: Jahn–Teller distortion promoted the forming of oxygen defects, resulting in optimal Mn *e*_g_ filling state and better electrical conductivity. e) Specific activities (current density at 1.6 V vs RHE) for SFO, CFO, CCFO, LMO, BSCF, and RuO_2_. Reproduced under the terms of the Creative Commons Attribution 4.0 International License.^[^ ^9^ ^]^ Copyright 2015, The Authors, published by Springer Nature. f) Electronic spin states of the octahedral site Co ions of ACoO_3_ (A = Ca, Sr) and Co_3_O_4_. Reproduced with permission.^[^ ^10^ ^]^ Copyright 2019, American Association for the Advancement of Science.

Several parameters are often used to describe the electrocatalytic performance of a catalyst, such as overpotential (*η*), Tafel Slope, exchange current density *(j*_0_), normalized activity, and stability (**Figure** **3**). These parameters can also provide insight into the thermochemical or kinetic barriers during OER.

#### 2.2.1 Overpotential (*η*)

Overpotential (*η*) is a common parameter used to describe the OER activity of catalysts,^[^ ^27^ ^]^ which is usually acquired from a linear sweep voltammetry (LSV) curve. The equilibrium potential for OER is 1.23 V versus RHE. The overpotential is defined by the difference between the applied potential (*E*) and the equilibrium potential (*E*_equ_), as shown in the following equation:

(11) $$\eta =E-E_{\mathrm{equ}}$$

Generally speaking, the value of the overpotential at a specific current density is selected for comparison. The potential for the onset of the OER current is defined as the onset potential *η*_0_, which represents the intrinsic OER activity, not influenced by the number of catalytic sites, the reactive kinetics, or any other factors. It only depends on the thermodynamic energy barrier of the catalysts. Nevertheless, it is difficult to determine precisely “the onset of OER current.” In general, two methods are adopted to define *η*_0_: it is settled either at a specified small current density (usually 0.5–1 mA cm^−2^), or by the intersection of the tangents of the baseline and the increasing current curve.

Moreover, the overpotential at the current density of 10 mA cm^−2^ (denoted as *η*_10_) is frequently used,^[^ ^28^ ^]^ because 10 mA cm^−2^ is the current density corresponding to the benchmark of 12.3% of the solar‐to‐fuel transfer efficiency under the irradiation of sunlight. Moreover, the overpotential at a large current density of 100 mA cm^−2^ (denoted as *η*_100_) is also chosen for some catalysts containing Fe, Co, and Ni elements in order to reduce the influence of the redox peak in the range of OER potential.

#### 2.2.2 Tafel Slope and Exchange Current Density (*j*_0_)

Tafel slope is an important descriptor for the reaction kinetics of OER.^[^ ^29^ ^]^ It is obtained by fitting the linear part of Tafel plot at the range of low current density, since the logarithm of current density and overpotential will diverge from the linear relationship at a high current density due to abundant gas bubbles. Tafel plot describes the relation of log(*j*) and *η*, both of which could be extracted from the LSV curve. Tafel slope is fitted by the equation as follows:^[^ ^29^ ^]^

(12) η=a+blog(j)

(13) $$a=2.303RT\log j_{0}/\alpha nF,\;b=2.303RT/\alpha nF$$

where *b* is referred to as the Tafel slope, *j*_0_ is the exchange current density, and *n* represents the number of transferred electrons in the OER reaction.

Notably, *α* is the charge transfer coefficient: the higher the value of *α*, the higher the charge transfer rate across the interface of the electrode and electrolyte. As can be seen from the equation, Tafel slope *b* is inversely proportional to *α*, which indicates that a lower Tafel slope corresponds to a higher reaction rate. In addition, the value of Tafel slope could be helpful to assess the possible reaction mechanism. According to the Butler–Volmer equation, the values of 29, 38, and 116 mV dec^−1^ correspond to the Tafel, Heyrovsky and Volmer rate‐determining step, respectively.^[^ ^30^ ^]^

The exchange current density (*j*_0_) is another parameter to assess the intrinsic catalytic activity of OER catalysts.^[^ ^31^ ^]^ *j*_0_ equals to the current density *j* when *η* = 0 in the Tafel equation. It represents the intrinsic capability of a catalyst to conduct the electrocatalytic OER reaction: a higher *j*_0_ means a greater OER activity of the catalyst.

#### 2.2.3 Normalized Specific Activity

The current density of OER can be normalized by geometric surface area of the working electrodes (disk surface area), the catalyst mass, and the catalyst surface area. Geometric current density is the most common parameter to evaluate the actual activity of the tested electrode and can provide practical guidance for full‐cell design. However, it is only applicable for the smooth and planar surface of electrodes.^[^ ^32^ ^]^ Due to the rough surface for most electrodes, it is equitable to compare the geometric activities for different catalysts. Mass‐normalized current density just considers the loading of catalysts, regardless of particle size, morphology, or other structure parameters. Hence, it is also not suitable to compare the OER activity for catalysts with different microstructures.

To exclude geometric effects and show the intrinsic activities of a catalyst, current density normalized by the catalyst surface area has been proposed. Brunauer–Emmett–Teller (BET) surface and electrochemically active surface area (ECSA) are two types of common measurements for the catalyst surface area. BET surface area normalized activity is mostly applied for porous materials. Nevertheless, the surface area obtained by N_2_ absorption and desorption test may not represent the electrocatalytic area, as electrolyte may not be able to access the micropores determined by the gas probe. ECSA is more representative to reflect the electrocatalytic activities of a catalyst and thus the ECSA‐normalized specific activity should be determined for the intrinsic OER activity irrespective of the loading and microstructure of catalysts. ECSA can be obtained from the double‐layer capacitance (*C*_dl_) method based on Equation (14)

(14) $$\mathrm{ECSA}=C_{\mathrm{dl}}/C_{s}$$

where *C*_s_ is referred as a general specific capacitance. *C*_dl_ is calculated from the CV curves with different scan rates based on Equation (15)

(15) $$j=\nu C_{\mathrm{dl}}$$

where *j* is the current in the middle of a CV potential range and *ν* is the scan rate. Plotting *j* as a function of *ν* yields a straight line with a slope equal to *C*_dl_. Notably, the potential window of CV should be selected without Faradaic current response.

#### 2.2.4 Faradaic Efficiency (FE)

Faradaic efficiency (FE) reflects the efficiency of utilization of the electrons in an electrochemical reaction.^[^ ^33^ ^]^ It is the ratio of the number of electrons used to generate a product over the total number of electrons supplied from the external circuit, i.e., the ratio of the amount of produced oxygen over the theoretical amount of oxygen in the OER case. FE is usually not 100% due to formation of by‐products, so it is a vital parameter to reflect the selectivity of a catalyst in general. The theoretical amount of oxygen could be calculated by Chronoamperometry (CA) or Chronopotentiometry (CP) measurements.^[^ ^34^ ^]^ The amount of produced oxygen could be obtained by many ways, such as water‐gas displacing method, gas chromatography or fluorescence spectroscopy. Another popular approach is through rotating ring‐disk electrode (RRDE) measurement, in which the catalyst is loaded on a glassy carbon disk, rather than on the Pt ring. The disk is subjected to a potential region of OER at a low scanning rate (5 mV s^−1^). In the meantime, a constant potential is applied on the ring that can reduce the evolved oxygen from the disk. The equation is as follows:

(16) $$\mathrm{FE}=I_{R}n_{D}/I_{D}n_{R}N_{\mathrm{CL}}$$

*I*_R_ and *I*_D_ are referred to as the current of ring and disk, respectively, *n*_R_ and *n*_D_ are the number of electrons transferred, respectively, both of which are 4 for OER. *N*_CL_ is the collecting efficiency of the ring electrode, which is determined by a ferro‐ferri redox probe method.^[^ ^35^ ^]^

#### 2.2.5 Stability

Stability of OER catalysts is of great significance for practical applications, particularly the long‐term endurability under relatively high current densities.^[^ ^33^ ^]^ The most common approach is long‐term cyclic voltammetry (CV), in terms of current or current density change after cycling. Moreover, CA or CP measurements are also frequently used to evaluate the OER stability. The current/potential for a stable catalyst can retain most of the original value after a long‐term test.

## 3 High Valence States for OER Based on AEM Pathway

### 3.1 High‐Valence Oxides (HVOs)

The valence state of a metal refers to the number of valence electrons involved the chemical bonding in a compound. Many elements have a fixed valence related to their position in the periodic table due to the octet rule while some others show multivalence, due to very similar energy levels of the valence electrons. For instance, Fe ion, unlike the divalent cation Ca^2+^, may exhibit +2, +3, and even +4. Oxidation states are usually represented by integers which may be positive, zero, or negative while in some cases, the average valence is statistically shown as a fraction, such as +3.5 for cobalt in Li_0.5_CoO_2_. The highest oxidation state was reported to be +9 in the tetroxoiridium (IX) cation (IrO_4_^+^). A high‐valence oxide usually means an enhanced valence (bonding) state of the metal, compared with that in its pristine oxide under standard conditions. For example, the common valence for Co is +2 and +3, thus oxides with average valence of cobalt greater than +3 are defined as HVOs, such as Sr_0.3_La_0.7_CoO_3_ (Co^3.3+^) and CaCoO_3_ (Co^4+^). Noble metal ruthenium and iridium oxides, as the benchmark OER catalysts, show a usual valence of +4, hence the Hg_2_Ru_2_O_7_ (Ru^5+^) is a high‐valence ruthenium oxide.

### 3.2 The *e*_g_‐Orbital Occupancy to Balance the Adsorption and Desorption Energy

The interaction between a catalyst and the reaction intermediates significantly influences the OER performance. According to the “Sabatier principle,” an ideal catalyst should bind the intermediates neither too strongly nor too weakly. Too strong a binding energy will hinder the desorption of the desirable product while too weak a binding energy will prevent the catalysts from capturing the reactants.^[^ ^25^ ^]^ Unfortunately, the binding energy between a catalyst and the intermediates is hard to evaluate experimentally. A more straightforward descriptor may be developed for the design of high‐performance OER catalysts. The filling of the *e*_g_ orbitals (i.e., the two d‐orbitals of higher energy than the three *t*_2g_ orbitals in TMs, due to degeneracy split) was noted to influence the binding of oxygen intermediates, and further determine the intrinsic OER activities of the oxides. The electronic configuration and the value of *e*_g_‐orbital occupancy (*e*_g_‐filling) can be deduced from the magnetic property, measured experimentally.^[^ ^10^, ^36^ ^]^

TMOs, as a coordination complex, is usually characterized by an octahedral structure with six ligand oxygen ions at the vertices and the metal ion in the center. According to crystal field theory, the valence electrons from the ligand oxygens will be closer to the metal *d*_z_^2^ and *d*_x_^2^_−y_^2^ orbitals and farther away from *d*_xy_, *d*_xz_, and *d*_yz_ orbitals, which results in the d‐orbitals splitting in energy.^[^ ^24^ ^]^ The three distal orbitals, *d*_xy_, *d*_xz_, and *d*_yz_ (collectively referred to as *t*_2g_), experience less repulsion and have lower energy than that of *d*_z_^2^ and *d*_x_^2^_−y_^2^ (collectively referred to as *e*_g_). TM *e*_g_ orbitals show a larger spatial overlap with the neighboring O 2p orbitals than the *t*_2g_ orbitals: the former forming stronger *σ*‐bonds and σ*‐antibonds, and the latter forming weaker *π*‐bonds and π*‐antibonds. The *σ*‐bonding *e*_g_ orbitals greatly influence the binding of oxygen intermediate species, and thus affect the OER performance. The optimized *e_g_*‐orbital occupancy for OER is close to unity because too low an *e*_g_ filling (*e*_g_ < 1) will result in too strong a binding to oxygen, while too high an *e*_g_ filling (*e*_g_ > 1) will lead to too weak a binding, neither of which favors the OER process. Shao‐horn's group systematically compared the relationship between the OER catalytic activity of various perovskite oxides and the occupancy of the *e*_g_‐symmetry electrons of the transition metals (Figure 3a). The activities show a volcano tendency with the optimal *e*_g_‐filling of Ba_0.5_Sr_0.5_Co_0.8_Fe_0.2_O_3–*δ*_ (BSCF) close to unity.^[^ ^14^ ^]^ The *e*_g_‐filling may serve as a valid descriptor for further design of OER catalysts with the optimal adsorption and desorption energy, to enhance OER intrinsic activity.

The oxidation states of metal ions significantly influence the electronic configurations. In general, a TM with a higher valence state possesses a larger crystal field splitting energy (Δ_0_), which prefers to occupy a low spin (LS) state. For instance, Co^2+^ in the CoO is usually in the high spin (HS) state (*t* ^5^ *e* ^2^) while Co^3+^ in the MCoO_3_ (M = La or Pr) is usually in the LS state (*t* ^6^ *e* ^0^).^[^ ^37^, ^38^, ^39^ ^]^ Interestingly, a deviation seems to exist with further increase of the valence state of Co to 4+. Co with a super‐high valence of Co^4+^ prefers to be in the intermediate spin (IS) or HS state with more electrons in the *e*_g_ orbitals, compared to Co^3+^ in the LS state.^[^ ^10^, ^37^, ^40^, ^41^, ^42^ ^]^ Similar results are also reported for the Fe‐based oxides.^[^ ^9^ ^]^ Such deviation is likely due to that the crystal field splitting energy and the pairing energy are compatible for the super‐high valence state. Recently researchers have demonstrated that increasing the valence is an effective strategy to optimize the *e*_g_‐filling of TMs for more efficient OER activities. For example, Zhao and co‐workers have developed a double perovskite PrBa_0.5_Sr_0.5_Co_1.5_Fe_0.5_O_5+*δ*_ nanofiber as a highly efficient OER catalyst.^[^ ^42^ ^]^ As shown in Figure 3b,c. All of the PrBa_0.5_Sr_0.5_Co_1.5_Fe_0.5_O_5+*δ*_ nanofibers with different diameters of 196, 83, and 20 nm (denoted as PBSCF‐I, PBSCF‐II, and PBSCF‐III, respectively) exhibit much better intrinsic OER activities than IrO_2_. The ultrafine PBSCF‐III nanofiber with the average valence of +3.28 shows the best intrinsic OER activity due to the optimized *e*_g_‐filling of 1.26. The bulk PBSCF‐0 possesses a higher valence of +3.36 and a larger *e*_g_‐filling of 1.36 shows a reduced intrinsic OER activity because of the weak interaction with the oxygen reactant. Moreover, Yuqiao and co‐workers optimized *e*_g_ electron filling and electronic conductivity of a high‐valence CaMnO_3_ at the same time for OER using only hydrogen treatment and rare earth metal Yb doping.^[^ ^43^ ^]^ Ca_0.9_Yb_0.1_MnO_3_ (denoted as CYMO‐P) possesses a nominal *e*_g_ filling of 0.1 after 10% Yb doping and further hydrogen treatment at different temperatures (320 °C: CYMO‐320, 350 °C: CYMO‐350) generates abundant oxygen vacancies, which further elevate the *e*_g_ filling. CYMO‐350 with a mixed valence of Mn^3+/4+^ owns the optimal *e*_g_‐filling of 0.8, which is close to unity, and exhibits the best OER activity (Figure 3d).

In addition to the mixed valence of oxides, oxides with Fe^4+^ and Co^4+^ have also been investigated. Yagi et al. have developed high‐valence perovskite oxides CaCu_3_Fe_4_O_12_ (CCFO) with the high spin Fe^4+^ (*t*_2g_^3^ *e*_g_^1^).^[^ ^9^ ^]^ CCFO exhibits higher OER catalytic activity (overpotential at onset potential: 0.31 V) than that of the state‐of‐the‐art OER catalysts such as BSCF and the benchmark RuO_2_ (Figure 3e). Moreover, owing to the strong covalent bonding network, CCFO exhibits a high OER stability over 100 cycles. Moreover, Xiang Li et al. have reported two isostructural ACoO_3_ (A = Ca, Sr) perovskites with Co^4+^ as high‐performance OER catalysts.^[^ ^10^ ^]^ An effective magnetic moment for ACoO_3_ was calculated as 4.1 *µ*_B_ by fitting with the Curie–Weiss law (Figure 3f) indicating the IS state of Co^IV^ (*t*_2g_^4^ *e*_g_^1^). For comparison, the spinel Co_3_O_4_ owns the Co^III^ ions with LS state (*t*_2g_^6^ *e*_g_^0^) and perovskite LaCoO_3_ processes mix spin Co^III^ ions with LS state (*t*_2g_^6^ *e*_g_^0^) at the ground state and HS (*t*_2g_^4^ *e*_g_^2^) at the excited state. High‐valence ACoO_3_ with the optimized *e*_g_‐filling exhibits a much higher OER activity than the low‐valence Co_3_O_4_ and LaCoO_3_.

### 3.3 Enhancing the Metal–Oxygen Covalency to Accelerate Charge Transfer in OER

The interaction between the transition metal d band and the oxygen p band plays an important role in the physical and (electro)chemical properties of TMOs, such as superconductivity and thermoelectricity, optical and magnetic behaviors, alkali metal intercalation voltages, and charge transfer during an electrocatalytic process.^[^ ^44^ ^]^ The interaction between the TM and adsorbed oxygen intermediates plays an important role in OER. Hence, it is insufficient to solely consider the electronic configuration of TM in the development of a good OER catalyst. Suntivich et al. further emphasize the correlation between the covalency of M–O and OER activity. Strong covalency between metal site and oxygen can promote the charge transfer between the active metal sites and the adsorbates.^[^ ^14^ ^]^

**Figure 4:**
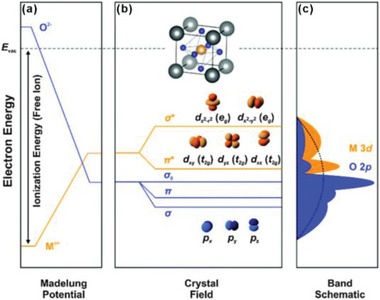
a) The energy of free ions in vacuum determined by their ionization energy/electron affinity; the on‐site Madelung potential of ions shifts these energies in the crystal lattice. b) Asymmetric covalent mixing between M 3d and O 2p orbitals form *σ*‐ and *π*‐bonding and antibonding orbitals (known as the “crystal field” interactions), with illustration of the M 3d and O 2p atomic orbitals—for octahedral coordination around a transition metal, the M 3d orbitals are split into *e*_g_ and *t*_2g_ states. c) Schematic diagram of the one‐electron band structure showing states with partial transition metal character (orange) and oxygen character (blue). Often, the three oxygen bands are shown as a single broad band indicated by the dashed curve. Reproduced with permission.^[^ ^24^ ^]^ Copyright 2015, Royal Society of Chemistry.

As the energy of an oxygen anion or a transition metal cation in vacuum is determined by the ionization energy/electron affinity. However, it will be inverted by the Madelung potential in the lattice of various crystal families, such as perovskite, spinel, rutile, and layered structures (**Figure** **4**a,b). Transition metal cations in a crystal lattice are coordinated with negatively charged oxygen anions, and the orbital energies of cations will rise because of the repulsive effect between electrons in the metal d band and the negative anions, and conversely the orbital energies of oxygen anions will decrease due to the attractive effect between the electrons in the O p band and the positive cations. Thus, transition metal d bands will be situated on the top of oxygen p bands with different degrees of spatial overlap (Figure 4c). The interaction between the metal (M) d orbitals and O 2p orbitals occur readily in the overlapped states of these bands due to the energetic compatibility. A larger overlap of metal (M) d orbitals and O 2p orbitals indicates a stronger covalency and an easier electron transfer between the two ions. In OER process, the metal d band usually serves as the redox center to donate electrons to the external circuit due to the higher energy level and then the electrons from the O 2p band will be transferred to refill the holes in the d band. Therefore, a fast charge transfer between the transition metal d band and the oxygen p band can accelerate the overall reaction. The overlap of these energy bands depends on many aspects, including the valence state of the cation, electronegativity of the ions, its bonding with the nearest‐neighbor, and the crystal structure, which influence the Madelung energy in the compounds. For a specific transition metal with the same crystal lattice, increasing the oxidation state will reduce the number of d electrons and move the metal d states closer to the O 2p states, increasing the covalency of metal–oxygen.

**Figure 5:**
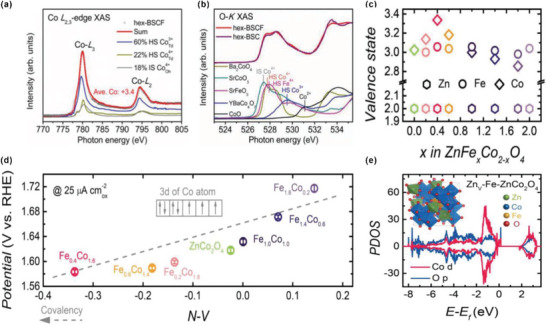
a) Simulated Co *L*_2,3_ XAS spectra of hex‐BSCF. b) Measured O‐K XAS spectra of hex‐BSC, hex‐BSCF, and several reference materials. Reproduced with permission.^[^ ^45^ ^]^ Copyright 2019, Wiley‐VCH. c) Valence states of Zn, Fe, and Co as a function of composition *x* in ZnFe_x_Co_2−x_O_4_ oxides. d) OER activity versus potential at 25 µA cm^−2^_ox_, as a function of “covalency” (the *N*–*V* parameter, see text). e) Computed partial electronic density of states (PDOS) of Zn_V_–Fe–ZnCo_2_O_4_. Reproduced with permission.^[^ ^46^ ^]^ Copyright 2018, Wiley‐VCH.

Recently, considerable efforts have been devoted to reveal how a strong covalency of the M—O bond in HVOs enhances the OER activity. For example, Zhu et al. have developed a hexagonally structured Ba_4_Sr_4_(Co_0.8_Fe_0.2_)_4_O_15_ (hex‐BSCF) as the ultrahigh OER catalyst with a low overpotential of only 340 mV at 10 mA cm^−2^ in 0.1 m KOH solution.^[^ ^45^ ^]^ The soft XAS with the surface sensitive total electron yield (TEY) has been applied to reveal the valence states and spin states of the TM. The average valence state of Co ions for hex‐BSCF can be calculated to be +3.4 (**Figure** **5**a). Furthermore, soft XAS measurement in the TEY mode at the O­K edge was conducted to investigate the degree of covalency of M—O (Figure 5b). With the increase of Co valence from CoO (+2) and YBaCo_4_O_7_ (+2/+3) to SrCoO_3_/Ba_2_CoO_4_ (+4), the pre­edge peak shifts to lower energies and the spectral weight becomes higher, indicating the enhanced Co–O covalency. The low energy O­K pre­edge peak (527.7 eV) of the hex­BSCF demonstrates the existence of high‐valence Co^4+^ with a strong Co–O covalency. Moreover, Zhou et al. have systematically explored the OER activity of a set of spinel oxides ZnFe_x_Co_2−x_O_4_ (*x* = 0–2.0). ZnFe_0.4_Co_1.6_O_4_ (the optimal) exhibits a higher OER activity than that of benchmark IrO_2_. The X‐ray absorption near‐edge spectroscopy (XANES) revealed that the valence of Fe remains at +3 and that of Co ranges from +2.86 to +3.34 for ZnFe_x_Co_2−x_O_4_ with different ratios of Fe. ZnFe_0.4_Co_1.6_O_4_ owns the highest Co valence of 3.34, indicating the existence of Co^4+^ (Figure 5c). The *N*–*V* parameter (*N* is the number of unpaired electrons of the Co atom, and *V* is the nominal valence state of the Co cation) can be used to evaluate the Co–O covalency. The lower value of *N*–*V* for TM means a higher ability to drag electronic density slightly from the oxygen atom, representing a greater TM‐O covalency. Figure 5d shows the relationship between *N*–*V* value and the OER activity of ZnFe_x_Co_2−x_O_4_, demonstrating that a stronger Co–O covalency results in a higher OER activity. Electronic density of states (DOS) calculations (Figure 5e) show that the Co 3d and O 2p centers get closer in V_Zn_–Fe–ZnCo_2_O_4_ than in ZnCo_2_O_4_, further confirming an enhanced Co–O covalency in V_Zn_–Fe–ZnCo_2_O_4_.

### 3.4 Reducing Band Gap to Improve Electrical Conductivity

Electrical conductivity is another crucial factor for OER because a high conductivity can facilitate the electron transportation between the surface of catalysts and the current collector and decreases the Ohmic potential drop and energy loss.^[^ ^43^ ^]^ Most of the oxides show a poor electrical conductivity because of high activation energy for electron transfer between cations. In order to overcome the poor transport property, oxides are usually dispersed in the conductive materials, such as carbon black, graphene, and Mxenes. or coated on a metal substrate. However, the conductive materials and substrates significantly increase the mass of electrode materials, and thus decrease the specific OER current density. Moreover, the carbon‐based materials are unstable at a high voltage and easily detach from the electrode, resulting in poor OER durability. Hence, it is desirable to develop intrinsic conductive catalysts for OER.

**Figure 6:**
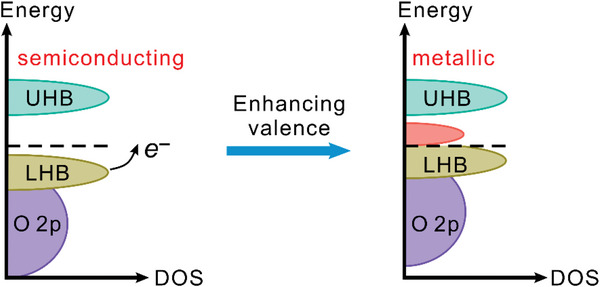
Schematic of the transition of the electronic structure (from semiconducting to metallic) via enhanced valence.

The electrical conductivities of materials are determined by the position of the valence band (VB) and conduction band (CB). The forbidden area between VB and CB is referred as the band gap, which is significantly influenced by the electron states near the Fermi level (*E*_F_). Partially filled energy bands near the Fermi level endow the materials metallic behavior while filled energy bands make them electronic insulators. TMOs, especially the Fe, Co, Ni‐based oxides, usually show poor electronic conductivities due to the large band gap, which impedes the charge transfer between the catalysts and the external circuit. Increasing the oxidation state of TMOs, a hole state is created accompanied by electron extraction from TM d band. The *E*_F_ shift down into the VB, leading to partially filled states near the Fermi level and thus the electronic conductivity increases (**Figure** **6**).

**Figure 7:**
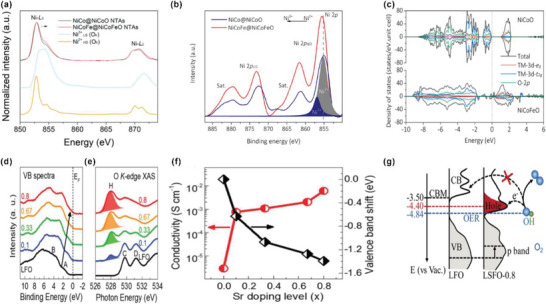
a) Comparison of Ni L‐edge XANES spectra of NiCo@NiCoO NTAs and NiCoFe@NiCoFeO NTAs to those of NiO (Ni^2+^_HS_, O_h_) and LaNiO_3_ (Ni^3+^_LS_, O_h_). b) XPS for Ni 2p. The shaded regions in (b) show the peak convolution areas from Ni species of different valence states. Ni/Co/Fe = 1:1:0.5. c) Calculated partial density of states (PDOSs) of bulk NiCoO and NiCoFeO. PDOSs above and below zero represent spin‐up and spin‐down states, respectively. The vertical dotted line represents Fermi energy level EF (set to zero). Reproduced with permission.^[^ ^47^ ^]^ Copyright 2019, American Chemical Society. d) VB XPS spectra and e) O K‐edge XAS of LSFO‐*x* (*x* = 0, 0.1, 0.33, 0.67, and 0.8). The VBM shows a gradual shift toward EF. The VBM is determined by linear extrapolation of the leading edge of the VBM to zero baseline intensity. e) The O K‐edge XAS show the development of a hole state at 528 eV. f) VBM shift values relative to the LFO VBM and electrical conductivity as a function of *x*. g) Experimentally measured occupied and unoccupied DOS near EF for LFO and LSFO‐0.8; the energy level is relative to the vacuum level (vs Vac.). Reproduced with permission.^[^ ^48^ ^]^ Copyright 2020, Royal Society of Chemistry.

Yan et al. reported that Fe substitution into the tetrahedral site of spinel NiCo_2_O_4_ (denoted as NiCoO) effectively enhances the OER activity.^[^ ^47^ ^]^ The Fe‐incorporated NiCo_2_O_4_ (denoted as NiCoFeO) exhibits outstanding OER performance with an ultralow overpotential of 201 mV at 10 mA cm^−2^ and a small Tafel slope of 39 mV dec^−1^, which is attributed to the optimized *e*_g_‐filling, increased degree of structure disorder, and higher electronic conductivity. X‐ray absorption fine structure (XAFS) and X‐ray photoelectron spectroscopy (XPS) are conducted to explore the valence states of the spinel oxides. As shown in **Figure** **7**a, with reference to spectra of high‐spine Ni^2+^ in NiO and low‐spin Ni^3+^ in LaNiO_3_, the characteristic peak of NiCoFeO shifts right to higher energy, indicating that NiCoFeO owns a higher average valence of Ni cation than NiCoO. The XPS results (Figure 7b) further confirm an increased ratio of Ni^3+^/Ni^2+^ on the surface of NiCoFeO superior to that of NiCoO (from 0.31 to 0.62). The total density of states (DOSs) of NiCoO and NiCoFeO from DFT calculation are compared in Figure 7c. Partially filled electronic states near the Fermi level can be observed in NiCoFeO due to the strong hybridization of TM 3d band and O 2p band, demonstrating a smaller band gap and higher electrical conductivity for NiCoFeO. Zechao et al. have also developed Sr‐doped LaFeO_3_ (LFO) with high electrical conductivity as high‐activity OER activity.^[^ ^48^ ^]^ The authors systematically explore how La^3+^ substitution with Sr^2+^ influence the electronic configuration and conductivity of LFO and further promote the OER. XPS and XAS, Figure 7d,e, are conducted to reveal the change of electronic structure with different ratio of Sr in LFO (denoted as LFO‐*x*). The VB XPS and O K‐edge XAS can be used to determine the occupied and unoccupied density of states (DOS), respectively. The electronic structure near the Femi level can be evaluated by the comprehensive results of XPS VB and O K‐edge XAS. As shown in Figure 7f, the occupied VB DOS gradually shifts to a lower binding energy with a higher Sr doping, which is attributed to the hole doping effect induced by Fe^4+^. Meanwhile, a new unoccupied state at 528 eV (hole state) gradually rises up with increasing Sr doping, which is induced by the oxidation state change of Fe from Fe^3+^ (*t*_2g_^3^ *e*_g_^2^) to Fe^4+^ (*t*_2g_^3^ *e*_g_^1^). The plot of the shift values of the valence band maximum (VBM) position toward *E*_F_ and electrical conductivities of LFO‐*x* versus the ratio of Sr doping (*x*) is shown in Figure 7g. That is, the hole in the valence band induced by the elevated valence state of Fe, decreases the band gap and enhances the intrinsic electrical conductivity of LFO‐*x*.

## 4 High‐Valence Activation of LOM, Breaking “Scaling Limitation” of AEM

### 4.1 Scaling Correlation via AEM Pathway

The reaction energy barrier (Δ*G*) for each transition step during OER via the traditional AEM pathway are described as follows^[^ ^49^ ^]^:

(17) $$\Delta G_{1}=\Delta G_{\mathrm{HO}*}-\Delta G_{H_{2}O\left(l\right)}-eU+k_{b}T\mathrm{In}a_{H^{+}}$$

(18) $$\Delta G_{2}=\Delta G_{O*}-\Delta G_{\mathrm{HO}*}-eU+k_{b}T\mathrm{In}a_{H^{+}}$$

(19) $$\Delta G_{3}=\Delta G_{\mathrm{HOO}*}-\Delta G_{O*}-eU+k_{b}T\mathrm{In}a_{H^{+}}$$

(20) $$\Delta G_{4}=\Delta_{O_{2}\left(g\right)}+\Delta G_{*}-\Delta G_{\mathrm{HOO}*}-eU+k_{b}T\mathrm{In}a_{H^{+}}$$

where the ΔG_*_, $\Delta G_{O_{2}\left(g\right)}$, and $\Delta G_{H_{2}O\left(l\right)}$ represent the free energy of the electrocatalyst, oxygen, and water molecule, respectively; the Δ*G*_HO*_, Δ*G*_O*_, and Δ*G*_HOO*_ represent the free energy of reaction intermediates with adsorbed groups of OH*, O*, and OOH*, respectively; $a_{H^{+}}$ represents the activity of the protons, *eU* represents the shift in electron energy, *k*_b_ is Boltzmann constant, *T* is temperature.

**Figure 8:**
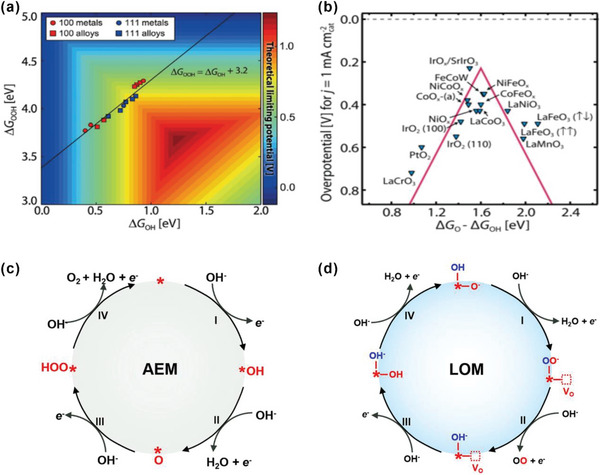
a) Theoretical limiting potential plot of ΔOOH* and ΔOH*. b) OER volcano plot for metal oxides. Reproduced with permission.^[^ ^52^ ^]^ Copyright 2017, American Association for the Advancement of Science. c) The classical four‐steps OER mechanism of proton‐coupled electron transfer (PCET), for which the rate‐determining step is often found to involve the formation of an *OOH intermediate, assisted by increasing the covalence of the M—O bond. d) Schematic of the lattice oxygen‐mediated mechanism. Reproduced under the terms of the Creative Commons Attribution 4.0 International License.^[^ ^53^ ^]^ Copyright 2022, The Authors, published by Springer Nature.

For an ideal OER catalyst, there should be no uphill energetics for each reaction step in the free energy diagram at the reversible electrode potential, and thus no overpotential is required to drive the catalysis. However, the thermodynamic approach has also revealed linearly correlation of the binding energies of the oxygen intermediates via the AEM pathway, often termed as the “scaling relationship.”^[^ ^50^, ^51^ ^]^ The energy difference between OOH* and OH* is demonstrated by many researchers to be of a fixed value: ≈3.2 ± 0.2 eV, for either metals or oxide surfaces because the catalytic site binds with both of HOO* and HO* via a single “M—O” bond coordination via the oxygen (**Figure** **8**a). Hence, the correlations between HOO* and HO* binding energies reduce the degrees of freedom which simplifies the activity description. The difference between Δ*G*_O*_ and Δ*G*_HO*_ (Δ*G*_O*_ – Δ*G*_HO*_) is commonly used as a universal descriptor to predict the OER activity since Δ*G*_1_ or Δ*G*_4_ rarely acts as the potential‐determining step. The overpotential can be expressed as

(21) $$\begin{array}{c} \eta^{\mathrm{OER}}=\left\{\max\left[\left(\Delta G_{O*}-\Delta G_{\mathrm{HO}*}\right),\;3.2\;\mathrm{eV}-\left(\Delta G_{O*}-\Delta G_{\mathrm{HO}*}\right)\right]/e\right\} \end{array}$$

Therefore, the plot of *η* ^OER^ as a function of (Δ*G*_O*_ – Δ*G*_HO*_) leads to a universal volcano relationship independent of the catalytic materials (Figure 8b), with a minimum overpotential of 0.37 eV at the reversible electrode potential.^[^ ^49^ ^]^

In order to bypass this scaling limitation and further enhance OER activities, it is important to break this single site “M‐O” coordination for the OER process. Several new OER mechanisms have been brought up, particularly the LOM, where lattice oxygen ligands are activated and serve as (part of) the redox center to donate directly electrons to the OER process with the holes left in the oxygen p band (anionic redox). This further facilitates the O–O coupling to generate the species (O_2_)^2−^, instead of the *OOH as with the AEM pathway. The schematic diagrams of two different OER pathway: AEM and LOM, are shown in Figure 8c,d. For a long time, the direct O–O coupling reaction was considered unfavorable because of the large activation barrier. However, an increasing number of OER catalysts with the LOM pathway has been reported recently, especially oxides with a high TM valence, a strong covalency M—O bond, a short O–O distance, and a large concentration of oxygen vacancies. Often several mechanistic changes are triggered by the tuning of one importance feature, such as the valency of the TM, to ensure a favorable OER pathway.

### 4.2 Triggering Lattice Oxygen as the Anionic Redox Center

**Figure 9:**
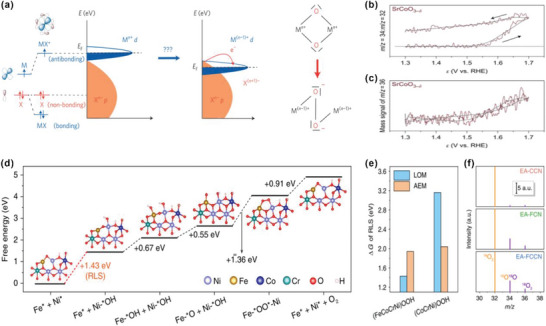
a) Triggering the anionic redox process in a solid. Schematic of the transition metal ligand (MX) band structure made from antibonding MX* states (described as d band), non‐bonding purely ligand X states (described as p band) and bonding MX states (this band is very low in energy and not involved in the redox reaction, it is therefore not represented for the sake of clarity). Reproduced with permission.^[^ ^55^ ^]^ Copyright 2016, Springer Nature. b,c) ^34^O_2_/^32^O_2_ ratios and ^36^O_2_ signal, where the straight lines correspond to the natural abundance of ^18^O of 0.2%. The arrows indicate forward and backward scans. Reproduced with permission.^[^ ^8^ ^]^ Copyright 2017, Springer Nature. d) Free energy diagram of OER cycling at Fe–Ni dual‐site on (FeCoCrNi)OOH model. e) The determined Δ*G* of RLS via LOM and AEM pathway in different models. f) The detected MS signals of generated oxygen molecule using ^18^O isotope‐labeled catalysts. The signals are normalized through initializing the intensity of ^16^O_2_ as 1000 a.u. Reproduced under the terms of the Creative Commons Attribution 4.0 International License.^[^ ^54^ ^]^ Copyright 2020, The Authors, published by Springer Nature.

The relative position of the TM d band and oxygen p band greatly influences the OER pathway. The energy of the TM d band is usually above the O p band, the metal sites will act as the redox center to donate during OER (**Figure** **9**a), which may be termed as the “cationic redox.” With the increasing oxidation state of TM, the TM d band shifts downward, and under a certain circumstance, the energy level of the d band is below the top of the O p band. Then the lattice oxygen can serve as the redox center to donate electrons to the external circuit, with the holes in the O p band, which may be termed as the “anionic redox.” The holes in the oxygen p band further facilitate the direct lattice O–O coupling to generate the species O_2_^−^, instead of the *OOH species in the AEM, effectively bypassing the scaling correlation. Alexis et al. report the direct evidence of the lattice oxygen activation in the OER of a high‐valence SrCoO_3−*δ*_ by means of the ^18^O isotope. In situ mass spectroscopy is used to evaluate the oxygen molecular weights generated during OER, where the mass‐to‐charge ratio *m*/*z* of 32, 34, and 36 represent ^16^O^16^O, ^16^O^18^O, and ^18^O^18^O, respectively, Figure 9b,c. The evident signal of ^16^O^18^O and ^18^O^18^O are detected when the applied voltage is higher than 1.5 V versus RHE for SrCoO_3−*δ*_, demonstrating the lattice oxygen involvement during OER. In contrast, only ^16^O^16^O is identified for the low‐valence LaCoO_3_, indicating no lattice oxygen activation.^[^ ^8^ ^]^ Ning et al. also demonstrated that high‐valence Ni site is energetically favorable for the LOM pathway when investigating the OER activity of the electrochemically activated alloys.^[^ ^54^ ^]^ The electrochemically activated FeCoCrNi alloy (EA‐FCCN) shows far better OER activity than that of FeCrNi (EA‐FCN) or CoCrNi (EA‐CCN), with the overpotential of 221 mV at 10 mA cm^−2^. According to the ex situ sXAS analysis, the Co^3+^ and Fe^3+^ are maintained during OER while Ni undergoes a dynamic oxidation to Ni^4+^ for the EA‐FCCN. The d band of Ni^4+^ downshifted into the O p band and thus lattice oxygens are activated as the redox center. It is demonstrated that oxides with a high‐valence Ni is more energetically favorable for LOM. DFT simulations are carried out to investigate the energy barriers for each step (Δ*G*) for both mechanisms. The Δ*G* of the rate‐determine step (RDS), i.e., the maximum ΔG, is determined for (FeCoCrNi)OOH and (CoCrNi)OOH, Figure 9d,e. Clearly, (FeCoCrNi)OOH shows a smaller Δ*G* of RDS via LOM than that via AEM. ^18^O‐isotope‐labeling mass spectrometry further confirms the Lattice oxygen activation process for the EA‐FCCN (Figure 9f).

### 4.3 Facilitated Deprotonation to Reduce Activation Energy

OER involves four‐electron, four‐proton transfer process (4‐PCET) and the total reaction is given as follows^[^ ^32^ ^]^:

(22) $$4{\mathrm{OH}}^{-}\to O_{2}+2H_{2}O+4e^{-}\;\left(\mathrm{alkaline}\;\mathrm{solution}\right)$$

(23) $$2H_{2}O\to O_{2}+4H^{+}+4e^{-}\;\left(\mathrm{acid}\;\mathrm{solution}\right)$$

**Figure 10:**
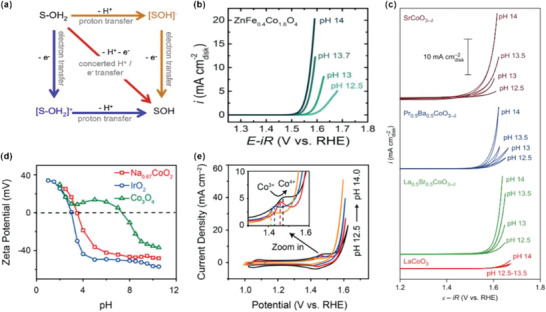
a) Square reaction scheme for deprotonation from the oxide surface (S) showing the sequential and concerted proton‐coupled electron transfer reactions. Reproduced with permission.^[^ ^56^ ^]^ Copyright 2016, Elsevier. b) CV of ZnFe_0.1_Co_1.6_O_4_ scanned in O_2_‐saturated KOH (pH = 12.5–14) at a scan rate of 10 mV s^−1^. Reproduced with permission.^[^ ^46^ ^]^ Copyright 2018, Wiley‐VCH. c) CV measurements from O_2_‐saturated 0.03 m KOH (pH 12.5) to 1 m KOH (pH 14) recorded at 10 mV s^−1^. Reproduced with permission.^[^ ^8^ ^]^ Copyright 2017, Springer Nature. The pH‐dependent OER behavior of Na_0.67_CoO_2_. d) Zeta potential of the catalysts. e) CV measurements of Na_0._67CoO_2_ in O_2_‐saturated KOH with pH 12.5–14. Inset shows the enlarged CV part from 1.3 to 1.6 V. Reproduced under the terms of PNAS license.^[^ ^36^ ^]^ Copyright 2019, National Academy of Sciences.

Typically, electrons and protons are transferred simultaneously during OER, which is referred to as a concerted proton–electron transfer (CPET) mechanism (red lines in **Figure** **10**a). The mechanism can keep the charge balance and don't generate any charged reaction intermediates, which are usually high in free energy diagram. However, due to diverse oxidation state of TM and flexible M—O bond, TMO can tolerate some degree of charge build‐up during the reaction and the decoupling of proton and electron transfer mechanism becomes possible (orange and blue lines in Figure 10a).^[^ ^56^ ^]^ The proton and the electron can transfer sequentially with a charged intermediate via these two routes. Because of the existence of the non‐concerted proton electron transfer step via LOM, a relatively fast deprotonation is desirable to break the traditional concerted proton–electron transfer step in AEM, which is favorable to enact the LOM.

The thermochemical or kinetic barriers of electron transfer and proton transfer are influenced by different factors. The former (electron transfer) is determined by the additional energy (the OER overpotential) along with the redox potential of the active sites and the potential difference between the three phases of solid/liquid/gas while the later (proton transfer) is associated with the acid dissociation constant (pK_a_) of catalyst and the pH of the electrolyte. If pH > pK_a_, deprotonation can occur spontaneously via a chemical step with zero free reaction energy. For catalysts with a low pK_a_, deprotonation is favorable in high pH solution and might occur before electron transfer while concerted proton–electron transfer happens in a low pH solution. Thus, they will exhibit pH dependent OER activities. Metal sites with a higher oxidation state will possess a greater electronegativity and thus a stronger attraction for the O‐2p electron, which will weaken the attraction of surface oxygen for the surface proton and make proton dissociation more easily via a chemical step.^[^ ^10^, ^36^ ^]^ Hence, HVOs usually show an easy deprotonation tendency and exhibit a pH‐dependent OER performance, which is also beneficial to activate the LOM pathway.

Goodenough et al. investigate the relationship between surface deprotonation and the surface charge density of oxides in the aqueous solution.^[^ ^57^ ^]^ When oxides are immersed into an aqueous solution, the proton of the “bound water” will come into equilibrium with the pH of solution with different charge on the surface. The pH of zero charge is defined as the point of zero‐zeta potential (pzzp). The oxides will accept proton to become positively charged at a pH < pzzp and donate proton to become negatively charged at a pH > pzzp. Therefore, pzzp is an effective descriptor to evaluate the ability of deprotonation for catalysts. In our previous work,^[^ ^36^ ^]^ we reported that Na_0.67_CoO_2_ possesses a much smaller pzzp (pH = 4) than that of spinel Co_3_O_4_ (pH = 7.5), indicating a high acidity of Na_0.67_CoO_2_ (Figure 10d). The onset potential and current density of Na_0.67_CoO_2_ at different pH values are compared in Figure 10e, demonstrating the pH‐dependent behavior on the RHE scale. Specifically, the oxidation voltage of surface Co^III^ to Co^IV^, accompanied with the electrochemical deprotonation at the first step, is reduced with the increase of pH because of the easier deprotonation process at a higher pH, Figure 8e. The strong covalence of the Co^III/IV^—O bond of Na_0.67_CoO_2_ weakens the attraction of surface oxygen for the surface proton and make deprotonation proceed more easily. Moreover, Alexis et al. found that La_0.5_Sr_0.5_CoO_3−*δ*_, Pr_0.5_Ba_0.5_CoO_3−*δ*_, and SrCoO_3−*δ*_ (Co^3+/4+^) show pH‐dependent OER behaviors (Figure 10c) on the RHE scale while LaCoO_3_ (Co^3+^) exhibits pH‐independent OER kinetics.^[^ ^8^ ^]^ Zhou et al. also report that ZnFe_0.4_Co_1.6_O_4_ (Co^+3.34^) shows the pH‐dependence performance.^[^ ^46^ ^]^ The pH dependence behavior indicates the existence of nonconcerted proton–electron transfer during OER, Figure 10b. Moreover, the two reports with nonconcerted proton–electron transfer during OER usually involves the redox of lattice oxygen, which will be introduced in the following chapter in details.

### 4.4 Effect of Oxygen Vacancy Concentration

**Figure 11:**
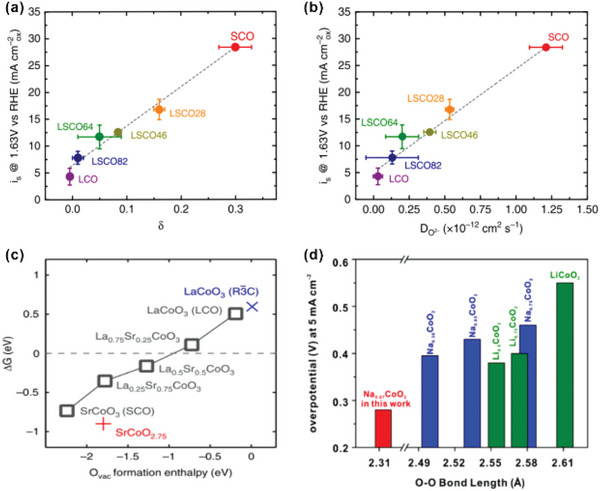
a) Correlation of oxygen evolution activity with the vacancy parameter d. The vacancy parameter is indicative of the underlying electronic structure where vacancies are generated when there is significant Co 3d and O 2p band overlap. b) Correlation of oxygen evolution activity with the oxygen ion diffusion rate, indicating that increased surface exchange kinetics trend with increased OER activity. Error bars represent standard deviation of triplicate measurements. c) O vacancy formation energy of the surface versus O vacancy formation enthalpy in the bulk. Reproduced under the terms of the Creative Commons Attribution 4.0 International License.^[^ ^19^ ^]^ Copyright 2016, The Authors, published by Springer Nature. d) OER performance (i.e., overpotential at 5 mA cm^−2^) versus the shortest O—O bond length in Na_x_CoO_2_ and Li_x_CoO_2_ with different Na and Li contents, respectively. Reproduced under the terms of PNAS license.^[^ ^36^ ^]^ Copyright 2019, National Academy of Sciences.

Oxygen vacancies usually play an important role in OER catalysis as it significantly influences the local electronic structure and surface chemistry. Mefford et al. demonstrate that an increase of oxygen vacancy concentration in Sr‐doped LaCoO_3_ facilitates the mobility of oxygen ions, effectively triggering an efficient LOM pathway to enhance the OER activity. Due to the lower valence of the Sr^2+^ ion than La^3+^, the overall charge neutrality of the compound is maintained by the elevation of Co valence and the generation of oxygen vacancies in La_1−x_Sr_x_CoO_3−*δ*_, i.e.:

(24) $$\begin{array}{c} {\mathrm{LaCo}}^{3+}O_{3}+x{\mathrm{Sr}}^{2+}-x{\mathrm{La}}^{3+}\to {\mathrm{La}}_{1-x}{\mathrm{Sr}}_{x}{\mathrm{Co}}_{1-y}^{3+}{\mathrm{Co}}_{y}^{4+}O_{3-\delta }+\frac{\delta }{2}O_{2} \end{array}$$

where *δ* is the amount of oxygen vacancies, and *y* is the amount of Co^4+^. As mentioned above, increasing the valence state of Co enhances the overlap between the Co d band and the O p band, facilitating OER to proceed via the “anionic redox.” Moreover, the existence of oxygen vacancies provides more reactive sites to bind OH* for further anionic redox reaction. High mobility of oxygen ions is indicative of fast oxygen diffusion, which also can facilitate the LOM pathway. As shown in **Figure** **11**a–c, the amount of oxygen vacancies and the mobility of oxygen ions scale with the level of Sr doping. Hence, a highly Sr‐doped LaCoO_3_ (*x* > 0.5) can catalyze water oxidation via the LOM while an insufficiently Sr‐doped LaCoO_3_ via AEM. The computed values for oxygen vacancy formation energy for La_1−x_Sr_x_CoO_3−*δ*_ also confirm that the elevated level of oxidation can decrease the formation energy of oxygen vacancies. Moreover, HVOs usually show a small oxygen separation, which promotes the direct O–O coupling. Moreover, Hao et al. systematically compare the relationship between the OER activities and the O—O bond length in layered Na_x_CoO_2_ and Li_x_CoO_2_ with different Na^+^ and Li^+^ contents. A shorter O—O bond length is beneficial to LOM and thus results in a better OER performance (Figure 11d).

## 5 Stability of HVOs

Apart from the activity, stability is another important performance indicator for an OER catalyst, especially for practical applications. As discussed above, there are two main OER mechanisms involving different intermediates and charge transfer pathways, which influence the stability of HVOs differently.

**Figure 12:**
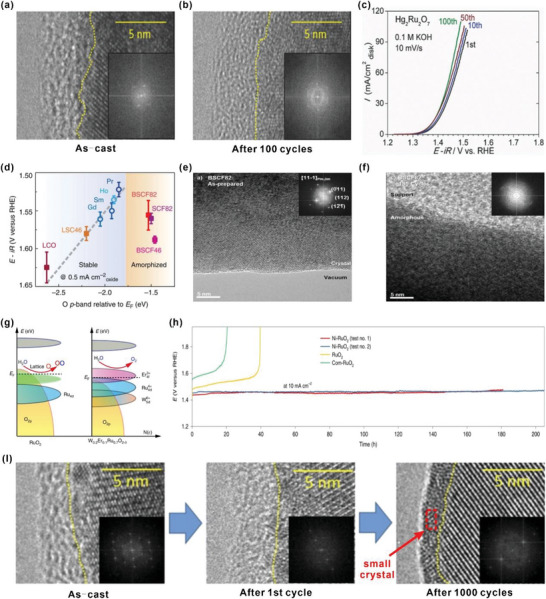
a,b) High‐resolution transmission electron microscopy (HRTEM) and fast Fourier transforms (FFTs) before and after OER tests for Hg_2_Ru_2_O_7_ (100 cycles). c) The linear sweep voltammograms (LSVs) of Hg_2_Ru_2_O_7_ at the scan rate of 10 mV s^−1^ for 1, 10, 50, and 100 cycles in 0.1 m KOH solution. Reproduced under the terms of the Creative Commons Attribution License.^[^ ^11^ ^]^ Copyright 2017, The Authors. d) evolution of the iR‐corrected potential at 0.5 mA cm^−2^_oxide_ versus the O p‐band center relative to E_F_ (eV) of (Ln_0.5_Ba_0.5_)CoO_3–*δ*_ with Ln = Pr, Sm, Gd, and Ho, for LaCoO_3_ (LCO), La_0.4_Sr_0.6_CoO_3–*δ*_ (LSC46), Ba_0.5_Sr_0.5_Co_0.8_Fe_0.2_O_3–*δ*_ (BSCF82), Ba_0.5_Sr_0.5_Co_0.4_Fe_0.6_O_3–*δ*_ (BSCF46) and SrCo_0.8_Fe_0.2_O_3–*δ*_ (SCF82). Reproduced with permission.^[^ ^13^ ^]^ Copyright 2013, Springer Nature. HRTEM of e) as‐prepared Ba_0.5_Sr_0.5_Co_0.8_Fe_0.2_O_3‐*δ*_ (BSCF82) powder and f) BSCF82 electrodes after 185 cycles (inset of (e) and (f): their corresponding FFTs). Reproduced with permission.^[^ ^59^ ^]^ Copyright 2012, American Chemical Society. g) Schematic diagrams of rigid band models for RuO_2_ and W_0.2_Er_0.1_Ru_0.7_O_2−*δ*−1_ in acidic OER. Reproduced under the terms of the Creative Commons Attribution 4.0 International License.^[^ ^60^ ^]^ Copyright 2020, The Authors, published by Springer Nature. h) Stability tests of Ni‐RuO_2_, RuO_2_ and Com‐RuO_2_. Reproduced with permission.^[^ ^61^ ^]^ Copyright 2022, Springer Nature. i) HRTEM and fast Fourier transform (FFT) images of as‐cast, after 1st and 1000 OER cycles for BaIr_0.8_Mn_0.2_O_3_. The boundaries between the crystalline layer and the amorphous layer are divided by yellow dotted lines. Reproduced with permission.^[^ ^62^ ^]^ Copyright 2022, Royal Society of Chemistry.

For the AEM pathway, the strong covalency of the M—O bond in HVOs not only promotes the charge transfer between the metal sites and the oxygen intermediates, resulting in a higher intrinsic activity, but also stabilizes the core crystalline structure, even at a high anodic potential, which endows the catalyst much enhanced OER stability. For instance, the surface of Hg_2_Ru_2_O_7_ (Ru^5+^) firstly undergoes rapid amorphization with an amorphous layer of 5 nm, which increases the electrochemically active surface area and thus enhances the OER current density slightly (**Figure** **12**a,b).^[^ ^11^ ^]^ However, the structure of the amorphous layer is usually unstable and too thick an amorphous layer usually degrades the OER stability. Due to the strong covalent bonding network formed by the Ru–O, the thickness of the amorphous layer for Hg_2_Ru_2_O_7_ remains nearly unchanged and no further erosion is observed in the core crystalline structure even after 100 CV cycles, which is the main reason for the outstanding OER stability (Figure 12c). A similarly enhanced stability was revealed in the CaCu_3_Fe_4_O_12_ with Fe^4+^.^[^ ^9^ ^]^

Moreover, the electron–electron correlation between metal–metal bond was also reported to play an important role in the OER activity and stability. For instance, Sr doping has been demonstrated to enhance the electron–electron correlation between Ru‐Ru 4d band in the CaRuO_3_, which not only enhances the initial OER activity, but also stabilizes the crystalline structure.^[^ ^58^ ^]^ The optimized oxide, Ca_0.9_Sr_0.1_RuO_3_, exhibits the strongest Ru–Ru electron correlation under *U*/*W* = 2.64 (where *U* and *W* denote the on‐site Coulomb potential and the bandwidth, respectively), which is demonstrated as the key factor to achieve both high activity and stability.

With further increase of the valency, the LOM pathway may be activated, which breaks the overpotential limitation induced by the “scaling” correlation, leading to more efficient OER. However, the cyclic “release and refilling” of the lattice oxygen species involved in the LOM pathway may lead to surface instability and eventually the collapse of the crystalline structure, degrading the stability of the catalysts. For instance, Ba_0.5_Sr_0.5_Co_0.8_Fe_0.2_O_3–*δ*_ (BSCF), with the O 2p band very close to the Fermi level, is reported to show the best OER intrinsic activity due to the activation of the LOM pathway. However, it undergoes a rapid and complete amorphization during OER, leading to poor stability (Figure 12d–f).^[^ ^59^ ^]^ The benchmark OER catalyst, RuO_2_ with high theoretical activity and relatively low cost, also suffers from poor operational stability, especially in an acidic solution, severely hindering its commercial application. Recently, it is reported that the instability of RuO_2_ in acidic media is mainly attributed to the intrinsic LOM pathway, which leads to the generation of the high‐valence soluble Ru^x+^ (*x* > 4) species (RuO_4_) at a relatively high applied voltage. A (W+Er) co‐doping strategy has been proposed to tune the electronic structure of RuO_2_, which increases the oxygen vacancy formation energy and suppresses the LOM pathway (Figure 12g).^[^ ^60^ ^]^ Moreover, Ni doping into RuO_2_ has also been demonstrated as an effective strategy to inhibit the LOM pathway and the developed Ni‐RuO_2_ exhibits excellent stability with very little current drop for more than 200 h in an acidic solution (Figure 12h).^[^ ^61^ ^]^

On the other hand, although a robust crystalline structure undoubtedly is favorable for stability, it usually leads to a poor intrinsic OER activity. More recently, partial substitution of the Ir‐site with Mn has been demonstrated as an effective strategy to enhance both the activity and stability of BaIrO_3_ in the acidic solution.^[^ ^62^ ^]^ It was revealed that Mn doping induced slight Ir dissolution due to competition for the same crystallographic sites, which facilitate the generation of a highly active amorphous layer with high‐valence Ir cations, leading to enhanced activity. Moreover, the appropriate balance between Ba‐ and Ir‐dissolution promote the formation of a short‐range ordered structure at the outermost surface, which prevents the lattice collapse and the excessive Ir dissolution in the core structure, resulting in the enhanced OER stability (Figure 12i). Hence, when the active LOM pathway is triggered for OER, a compensative effect is necessary to balance the stability, e.g., by substitutional partial dissolution of surface metallic species to raise the valence states of the remaining surface species, which seems to confine the active sites within the semi‐amorphous surface layer without degrading the core structure (Figure 12i).

**Figure 13:**
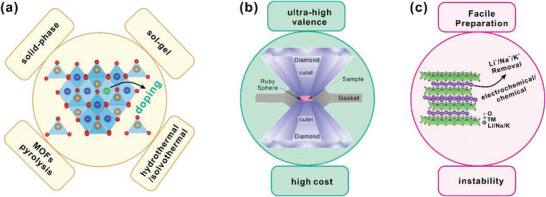
Schematic of preparation methods for high‐valence oxides: a) element doping; b) high pressure and high temperature via diamond anvil; and c) electrochemical/chemical delithiation/desodiation/depotassiation.

## 6 Synthesis Strategies for HVOs

### 6.1 Valence Tuning by Elemental Doping

With the flexible changes of compositions and crystal configuration for TMOs, doping is an effective way of regulating the electronic configuration, surface adsorption energy and electrical conductivity of TMOs. Most of the reported HVOs are synthesized via the strategy of elemental doping. Generally speaking, foreign metal ions with similar ionic radii but different valence states, or electronegativities, are chosen as dopants to engineer the physical and (electro)chemical properties of the parent material structure and to promote the OER performance. Nearly all common synthetic methods, including solid‐state, sol–gel, hydrothermal, electrospinning methods and pyrolysis of MOFs, can be utilized for elemental doping and the specific approach depends on the targeted dopants and lattice energies. The most common synthesis methods are discussed as follows (**Figure** **13**a).

#### 6.1.1 High‐Temperature Solid‐State Syntheses

The traditional high‐temperature solid‐state synthesis is the most common and effective method to prepare a diverse range of oxides. Generally speaking, the corresponding metals, metal (hydr)oxides, or metal compounds (such as carbonates, nitrates, sulfates, and acetates) with the stoichiometric ratio are mixed thoroughly as the raw materials, which are then thermally decomposed to form the targeted oxides. The refined crystalline structure without any impurity is also desirable for investigation of the OER mechanism from physical and chemical characteristics, such as crystalline structure, electronic configuration, and reaction pathways. Furthermore, the high yield of this method is useful for large‐scale commercial applications. For instance, Shao‐horn's group systematically compare the OER performance of Fe/Co/Ni‐based perovskite oxides, prepared via solid‐phase methods.^[^ ^8^, ^13^, ^14^ ^]^ Goodenough's group also synthesize distorted Na_0.67_CoO_2_ with “super short” oxygen separations via repeated solid‐phase annealing.^[^ ^36^ ^]^

However, due to relatively sluggish diffusivity in the solid state, rigorous reaction conditions are usually required, such as long reaction time and high annealing temperature, which greatly increase the cost of the catalysts. Moreover, the large particles induced by high temperature annealing is not suitable for OER. Ball milling is demonstrated as an alternative approach to reduce the reaction time and particle size and to generate a wide range of meta‐stable structures for further assessment. This technique is relatively under‐explored for OER catalysts.

#### 6.1.2 Sol–Gel

Sol–gel is another facile method to reduce the particle size of oxides down to the nanometer range. Typically, metal salts, together with organic chelating agents, e.g., citric acid, ethylene glycol, and glacial acetic acid, are uniformly mixed in a solvent (water or organic solution) at ambient conditions, which will go through a typical reaction, such as hydrolysis and condensation, to form a stable sol system. Then the sol will gradually lose the solvent to generate the gel. Finally, the nano‐scale oxides can be obtained after high temperature calcination of the gel, for a much shorter period of time than the solid‐state method to preserve the fine structure. For instance, high performance spinel oxides, ZnFe_x_Co_2−x_O_4_,^[^ ^46^ ^]^ ZnFe_2–x_Cr_x_O_4_,^[^ ^63^ ^]^ and CoAl_2_O_4_,^[^ ^64^ ^]^ are synthesized by Xu's group via the sol–gel route. Zhang et al. also successfully produce Sr‐doped pyrochlore‐structureY_2_Ru_2_O_7_ with the high‐valence Ru^4+/5+^ via this method.^[^ ^65^ ^]^

#### 6.1.3 Hydrothermal/Solvothermal

The hydrothermal method is a popular aqueous solution synthesis approach at temperature and high vapor pressure to develop controlled morphologies of nano‐/micro‐structured oxides, such as nanowires, nanofibers, nanotubes, microspheres, or oxide‐incorporated porous structures.^[^ ^66^, ^67^, ^68^ ^]^ The morphologies can be tailored by the reaction conditions, such as temperature, the choice of solution and the pH, and the templates with due consideration of the phase structures of the oxides. TM hydroxides/layered double hydroxide (LDH),^[^ ^69^, ^70^, ^71^ ^]^ are often prepared hydrothermally. Nevertheless, oxides with high crystallization point, such as perovskites, spinels, pyrochlores, and rutiles, are usually obtained with a further calcination step to ensure desirable structures.^[^ ^72^, ^73^, ^74^ ^]^

#### 6.1.4 Controlled Pyrolysis of Metal−Organic Frameworks (MOFs) and Biomass Structures

Due to the reticular porous structure and tunable functionality, MOFs and their derivatives attract much attention for energy storage and conversion.^[^ ^75^ ^]^ Biomass offers an environmentally benign and sustainable route for potentially large‐scale synthesis of functional porous structures. Controlled pyrolysis or partial pyrolysis of MOFs and biomass has been demonstrated as an effective way to prepared oxides and various nano‐structures with carbon coating or support.^[^ ^76^, ^77^ ^]^ The tunable and flexible metal sites are accessible for a wide range of metals, which make elemental doping feasible by regulating the metal ion in the MOF nodes.^[^ ^78^ ^]^ The morphology, porosity and chemistry of the derived structure can be maintained or tailored by reaction conditions, to create or expose more catalytic sites and to facilitate the mass transportation in OER. For example, Chen's group develop Mn/Cu‐doping RuO_2_ as high‐performance OER catalysts in an acidic solution with corresponding MOFs as precursors.^[^ ^79^, ^80^ ^]^ Moreover, Hao et al. also co‐dope Pt and La into the lattice of IrO_2_ to increase the OER activity and stability in an acidic solution with success.^[^ ^81^ ^]^

### 6.2 HVOs via High Pressure and High Temperature Synthesis

With the development of high‐pressure science, many of the targeted oxides that are inaccessible under ambient conditions, can be obtained using ultra high pressures (2–20 GPa) and high temperatures (800−2200 °C) (Figure 13b). It is demonstrated that the large lattice contraction under high pressure may induce the valence transition of compounds. It provides a new synthesis route for high‐valence oxides. Recently, a high pressure of 100 GPa has been achieved with specialized facilities, such as multi‐anvil presses (MAPs) and diamond anvil cells (DACs). Clearly, the oxides must remain kinetically stable after decompression back to ambient and at catalytic conditions, even though the high pressure is crucial for synthesis. Indeed, only a few of high‐pressure phases can remain intact when the pressure and temperature are quenched, such as diamond.

A few stable HVOs have recently been synthesized via high‐pressure and high‐temperature diamond‐anvil method, which exhibit outstanding OER performance as expected. For instance, a Fe^4+^‐based quadruple perovskite CaCu_3_Fe_4_O_12_ has been prepared under 1000 °C and 7 GPa,^[^ ^9^ ^]^ and notably, the compound shows better OER performance than the state‐of‐the‐art RuO_2_. The covalent bonding networks incorporating multiple Cu^2+^ and Fe^4+^ ions significantly enhance the structural stability of CaCu_3_Fe_4_O_12_, which is key to achieving highly active and durable catalysts. Shigeto et al.^[^ ^11^ ^]^ have reported Hg_2_Ru_2_O_7_, prepared under 950 °C and 6 GPa. This oxide shows much better OER activity than RuO_2_, with an ultra‐low overpotential of 150 mV at 10 mA cm^−2^. This outstanding OER performance can be explained in terms of the coexistence of the localized d‐bands and the metallic state in the structure. These findings indicate that the principles of non‐Fermi liquids could serve as new design criteria for highly active OER catalysts. Moreover, Xiang et al. synthesized highly active CaCoO_3_ and SrCoO_3_ catalysts with metallic conductivity under 1200 °C and 7 GPa.^[^ ^23^ ^]^ Both CaCoO_3_ and SrCoO_3_ show excellent OER performance compared with LaCoO_3_. CaCoO_3_ exhibits excellent OER activity with an onset potential of 1.48 V and a small overpotential of 260 mV at 10 mA cm^−2^. CaCoO_3_ also present a remarkable stability with 89% current density retention after a stability test for 50 000 s. The Co—O bond lengths of the perovskites CaCoO_3_ and SrCoO_3_ are much shorter than those of LaCoO_3_ and Co_3_O_4_ with Co^3+^, and then the stronger covalency of the Co—O bond contributes to the remarkable OER activity of these two Co^4+^‐based oxides. All of the above can demonstrate that high‐pressure synthesis is a viable method for developing the ambient‐pressure stabilized oxides as high‐valence OER catalysts.

### 6.3 Layered Oxides after De‐Lithiation/De‐Sodiation

Because of the special sandwich structure of the layered oxides, the alkaline metals (Li/Na/K) can be readily removed from the crystalline structure by chemical or electrochemical means to force the transition metal to a higher valence (Figure 13c). It is a very popular method to prepare some layered OER catalysts with high valence.

Lu et al.^[^ ^21^ ^]^ have developed a method for electrochemical lithium tuning of catalytic materials in an organic electrolyte for subsequent enhancement of the catalytic activity in aqueous solutions. By continuous extraction of lithium ions out of LiCoO_2_, a popular cathode material in lithium‐ion batteries, in an organic electrolyte, a Li_0.5_CoO_2_ structure is formed, which shows significantly improved catalytic activity for OER. This enhancement is ascribed to the unique electronic structure of the delithiated compound. This methodology is also proved in several mixed metal oxides (LiCo_0.5_Ni_0.5_O_2_, LiCo_0.5_Fe_0.5_O_2_, LiCo_0.33_Ni_0.33_Fe_0.33_O_2_, LiCo_0.33_Ni_0.33_Mn_0.33_O_2_, etc.) with similar positive effect. Particularly, the electrochemically delithiated LiCo_0.33_Ni_0.33_Fe_0.33_O_2_ demonstrate a remarkable OER activity with a low onset potential of 1.47 V and an overpotential of 295 mV at 10 mA cm^−2^, better than the benchmark iridium/carbon catalyst. Pearce and co‐workers^[^ ^82^ ^]^ have also synthesized fully delithiated *β*‐IrO_3_ by electrochemical oxidation/delithiation of *β*‐Li_2_IrO_3_ in Li‐ion batteries, demonstrating the high activity and power stability of iridium‐based OER catalysts from the formation of a high‐valence IrO_x_ intermediate. The as‐prepared *β*‐H_2_IrO_3_ by the hydrothermal ion exchange of *β*‐Li_2_IrO_3_ in an acid solution shows enhanced OER activity and stability, outperforming the IrO_2_ catalyst.

Maiyalagan et al.^[^ ^22^ ^]^ have also synthesized Li_0.5_CoO_2_ with a chemical delithiation method using NO_2_BF_4_ as an oxidant, for enhanced OER activity. The extraction of lithium from LiCoO_2_ results in further oxidation of some Co^3+^ ions to Co^4+^ ions and creation of mixed‐valent Co^3+/4+^ ions. This leads to a strong covalency as a result of a larger overlap between the Co^3+/4+^:3d and the O^2−^:2p states. Along with the high electronic conductivity arising from the partially filled Co^3+/4+^, the compound can accelerate the OER process. Yan's group^[^ ^83^ ^]^ have used I_2_ in an acetonitrile solution as an oxidant to extract Na from NaNi_y_Fe_1‐y_O_2_ to form layered Na_1−x_Ni_y_Fe_1−y_O_2_ double oxide electrocatalysts with Ni^3+/4+^. Notably, Na_0.08_Ni_0.9_Fe_0.1_O_2_ just only requires a low overpotential of 260 mV to achieve 10 mA cm^−2^, and it also shows excellent stability after a test for 70 h, superior to the state‐of‐the‐art noble metal‐based oxides and layered double hydroxide catalysts, such as RuO_2_ or FeNi(OH)_x_. Our group^[^ ^36^ ^]^ also successfully produce a layered‐oxide Na_0.67_CoO_2_ with low‐spin Co^III/IV^ ions (Co^III^: π*^6^ σ*^0^; Co^IV^: π*^5^ σ*^0^) by the solid‐state reaction with repeated annealing. The prepared catalyst shows an outstanding OER activity with an onset potential of 1.5 V versus RHE and a small overpotential of 290 mV at 10 mA cm^−2^. The high activity is attributed to the unusually short O–O separation that favors the formation of peroxide ions by O^−^–O^−^ interactions, followed by O_2_ evolution in preference to the conventional route via surface O–OH^−^ species. A strong hybridization of the O–2p and the low‐spin Co^III^/Co^IV^ *π*‐bonding d states is the other important factor for the ultrafast oxygen evolution reaction.

## 7 Recent Advances in HVOs with Different Types of Structures

**Figure 14:**
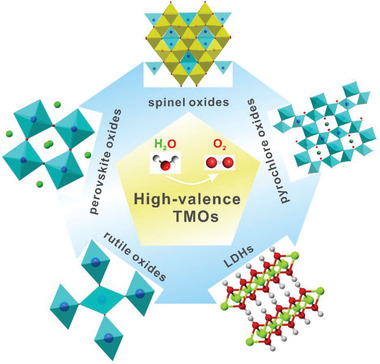
Crystal structures of common HVOs for OER, including perovskites, spinels, pyrochlores, rutiles, and LDHs.

HVOs are a large family of OER catalysts. Here we mainly discuss several representative structures, including high valent perovskite, spinel, pyrochlore, rutile oxides, and TM hydroxides (**Figure** **14**). All HVOs discussed in this review are summarized in **Tables** 1, 2, 3, 4, 5.

**Table 1:** OER performance, mechanism, and synthesis of high‐valence perovskite oxides

No	Catalyst	Active metal sites	OER activity	Durability	Electrolyte	Mechanism	Synthesis method
1	La_0.5_Sr_0.5_NiO_3_^[^ ^88^ ^]^	Ni^3+/4+^	290 mV @50 µA cm^−2^	–	0.1 m KOH	O p band shift up to *E*_F_; facilitate the charge transfer; strong Ni 3d‐O 2p hybridization	Oxygen plasma‐assisted molecular beam epitaxy (OPA‐MBE)
2	SrCoO_2.7_^[^ ^19^ ^]^	Co^3.4+^	400 mV @28.4 mA cm^−2^_ox_	24 h @ 10 A g^−1^_ox_	0.1 m KOH	Increase oxygen vacancy; promote LOM	Reverse‐phase hydrolysis approach with a post‐annealing
3	Pr_0.5_Ba_0.5_CoO_3_^[^ ^13^ ^]^	Co^3+^ (IS), Co^4+^ (HS)	220 mV (*η*_0_)	2 h @ 5 mA cm^−2^	0.1 m KOH	O p‐band center close to the *E*_F_; *e*_g_‐filling close to unity	Solid‐state route
4	Sr_0.9_Na_0.1_RuO_3_^[^ ^89^ ^]^	Ru^4+/5+^	170 mV @10 mA cm^−2^	20 cycles	0.1 m HClO_4_	Optimize the adsorption energies of intermediates	Sol–gel chemistry followed by thermal treatment
5	SrCoO_3–*δ*_^[^ ^8^ ^]^	Co^4+^	320 mV @ 3.5 mA cm^−2^	–	1 m KOH	Lattice‐oxygen oxidation	Solid‐state route
6	CaCoO_3_^[^ ^10^ ^]^	Co^4+^	260 mV @ 10 mA cm^−2^	20 000 s @ 1.6 V	0.1 m KOH	Shorter surface oxygen separation; Chemical deprotonation; LOM	High‐pressure solid‐state reaction
7	Sr_2_Co_1.5_Fe_0.5_O_6–*δ*_^[^ ^91^ ^]^	Co^3+/4+^	318 mV @ 10 mA cm^−2^	10 h@ 1.55 V	1 m KOH	Layered oxygen‐deficient; strong covalence of Co–O	Sol–gel followed by annealing
8	LaCo_0.9_Fe_0.1_O_3_^[^ ^92^ ^]^	Co^3+^ (HS)	400 mV @ 0.272 mA cm^−2^_oxide_	–	1 m KOH	Increased HS Co3+ ratio; strong covalence of Co–O	Sol–gel and a post‐calcination
9	LaNi_0.9_Cu_0.1_O_3_^[^ ^93^ ^]^	Ni^3+^	–	–	0.1 m KOH	Abundant lattice strains and oxygen vacancies	Hydrothermal method
10	Ba_0.5_Sr_0.5_Co_0.8_Fe_0.2_O_3–*δ*_^[^ ^14^ ^]^	IS Co^2.8+^	250 mV @ 50 µA cm^−2^_ox_	–	0.1 m KOH	*e*_g_‐filling close to unity; strong M‐oxygen covalency	Co‐precipitation with a post‐calcination method
11	La_0.2_Sr_0.8_Co_0.8_Fe_0.2_O_3–*δ*_^[^ ^20^ ^]^	Co^3+^	310 mV @ 10 mA cm^−2^	500 cycles	0.1 m KOH	Surface reconstruction to form Co oxy(hydroxide) layer	Flame spray synthesis
12	PrBa_0.5_Sr_0.5_Co_1.5_Fe_0.5_O_5+*δ*_^[^ ^42^ ^]^	Co^3+^ (IS) and Co^4+^ (HS)	358 mV @ 10 mA cm^−2^_disk_	12 h @10 mA cm^−2^_disk_	0.1 m KOH	A stronger adsorption of oxygen adsorbates; efficient charge transfer between Co and O	Electrospinning method followed by calcination process
13	La_0.6_Ca_0.4_Fe_0.7_Ni_0.3_O_2.9_^[^ ^94^ ^]^	Ni^3.4+^	380 mV @ 400 A g^−2^_ox_	10 h @ 10 A g^−2^_ox_	1 m NaOH	Activate LOM	Ultrasonic spray pyrolysis method with a post‐calcination
14	La_1.4_Sr_0.6_NiMoO_6_^[^ ^95^ ^]^	Ni^2+/3+^	367 mV @ 1 mA cm^−2^	–	1 m KOH	Reduce the Schottky barrier for electron transfer	Sol–gel method with a following annealing

**Table 2:** OER performance, mechanism, and synthesis of high‐valence spinel oxides

No	Catalyst	Active metal sites	OER activity	Durability	Electrolyte	Mechanism	Synthesis methods
1	Zn_0.75_Co_2.25_O_4_^[^ ^97^ ^]^	Co^2+/3+^	320 mV @ 10 mA cm^−2^	7500 s @ 1.63 V	1 m KOH	Shorter length of Co^3+^–O; facilitate the formation of Co^4+^; promote the deprotonation of OOH species	Hydrothermal method
2	Li_0.5_Zn_0.5_Fe_0.125_Co_1.875_O_4_^[^ ^64^ ^]^	Co_oh_^3+^ (LS)	350 mV @ 50 µA cm^−2^_ox_	–	1 m KOH	Strong Co_oh_–O interaction	Nitrate decomposition method with a post‐annealing
3	ZnFeCrO_4_^[^ ^63^ ^]^	HS Fe_oh_^3+^, Cr _oh_^3+^	450 mV @ 50 µA cm^−2^_ox_	–	1 m KOH	Optimize *e*_g_ filling; optimize the hybridization degree of the TM_oh_ 3d‐O 2p states	Sol–gel method with a post‐annealing
4	CoFe_0.25_Al_1.75_O_4_^[^ ^100^ ^]^	Co^3+^	310 mV @ 50 µA cm^−2^_ox_	48 h @ 20 µA cm^−2^_ox_	1 m KOH	Facilitate surface reconstruction into active Co oxyhydroxides; activate deprotonation on Co oxyhydroxides	Sol–gel method following calcination
5	Mn_3_O_4_^[^ ^101^ ^]^	Mn^3+^	300 mV @ 25 µA cm^−2^_ox_	–	0.1 m KOH	Optimize *e*_g_ occupancy of the active cation on the octahedral sites	Solid‐state chemistry method with a post‐calcination
6	CoFe_1.7_Ni_0.3_O_4_^[^ ^98^ ^]^	Co^2+/3+^	430 mV @ 1 mA cm^−2^	–	1 m KOH	Increased Co^2+^/Co^3+^ pair in the octahedral site	Ceramic powder method
7	NiCo_2_O_4_^[^ ^72^ ^]^	Ni^3+^/Co^3+^	400 mV @ 0.32 mA cm^−2^_BET_	–	1 m KOH	Enhance the hybridization of Ni/Co 3d‐O 2p; Ni^3+^ induced hole states reduce the energy barrier for electron transfer; reduce the adsorption of OH intermediate	Hydrothermal method with a post‐annealing
8	LiCoVO_4_^[^ ^99^ ^]^	HS Co_oh_^2+^	290 mV@ 1 mA cm^−2^_ox_	–	1 m KOH	Stronger Co—O bond covalency, active sites in magnetically polarized channels facilitate the extraction of certain spin‐oriented electrons from the singlet reactants	Solid‐state synthesis method

**Table 3:** OER performance, inner mechanism, and synthesis of high‐valence pyrochlores

No	Catalyst	Active metal sites	OER activity	Durability	Electrolyte	Mechanism	Synthesis method
1	Y_1.85_Zn_0.15_Ru_2_O_7–*δ*_^[^ ^103^ ^]^	Ru^4+/5+^	291 mV @ 10 mA cm^−2^	2000 cycles	0.5 m H_2_SO_4_	Improve the electrical conductivity	Sol–gel method with a post‐calcination
2	Y_1.7_Sr_0.3_Ru_2_O_7_^[^ ^65^ ^]^	Ru^3.97+^	264 mV @ 10 mA cm_geo_^−2^	28 h @ 10 mA cm_geo_^−2^	0.5 m H_2_SO_4_	High electrical conductivity; Strong Ru–O to promote charge transfer; lower the energy barrier of the overall reaction	Sol–gel method with a post‐annealing
3	Hg_2_Ru_2_O_7_^[^ ^11^ ^]^	Ru^5+^	150 mV @ 10 mA cm^−2^	100 h @ 1.5 V	0.1 m KOH	Coexistence of the localized d‐bands and the metallic state	High‐pressure solid‐state synthesis
4	Y_2_Ru_1.6_Y_0.4_O_7–*δ*_^[^ ^107^ ^]^	Ru^4+/5+^	270 mV @ 18.1 mA cm^−2^	–	0.1 m HClO_4_	Optimized energy band structure owing to the oxygen lattice defects and mixed oxidation state of Ru^4+/5+^	Sol–gel method with a post‐calcination
5	Y_2_Ru_1.9_Mn_0.1_O_7–*δ*_^[^ ^108^ ^]^	Ru^4+/5+^	273 mV @ 10 mA cm^−2^	11 h @ 10 mA cm^−2^	0.5 m H_2_SO_4_	More oxygen vacancies and strong covalence of Ru–O	Sol–gel method with a post‐calcination

**Table 4:** OER performance, mechanism, and synthesis of high‐valence LDHs

No	Catalyst	Active metal sites	OER activity	Durability	Electrolyte	Mechanism	Synthesis method
1	Gelled FeCoW OOH^[^ ^111^ ^]^	Co^2+/3+^/Fe^2+/3+^	191 mV @ 10 mA cm^−2^	550 h @ 30 mA cm^−2^	1 m KOH	Optimize adsorption energies for OER intermediates	Sol–gel method and a post‐refluxing method
2	CoFeCr LDH/NF^[^ ^71^ ^]^	Co^2+^/Fe^3+^	202 mV @ 10 mA cm^−2^	20 h @ 10 mA cm^−2^	1 m KOH	Stabilize Co active sites in a high oxidized state; reduce the energy barrier of H_2_O adsorption	Hydrothermal method
3	NiFeV LDHs^[^ ^69^ ^]^	Fe^3+^	195 mV @ 20 mA cm^−2^	18 h @ 1.48 V	1 m KOH	Modify the electronic structure; Narrow the bandgap and enhance electric conductivity; Facilitate electron transfer	Hydrothermal method
4	Ni_3_FeAl_0.91_ LDHs/NF^[^ ^70^ ^]^	Ni^3+^/Fe^3+^	304 mV @ 20 mA cm^−2^	18 h @ 20 mA cm^−2^	1 m KOH	Low‐coordinated Ni and Fe atoms and defects	Hydrothermal method

**Table 5:** OER performance, mechanism, and synthesis of high‐valence rutiles

No	Catalyst	Active metal sites	OER activity	Durability	Electrolyte	Mechanism	Synthesis method
1	Cu doped RuO_2_^[^ ^80^ ^]^	Ru^4+^	188 mV @ 10 mA cm^−2^	8 h @ 10 mA cm^−2^	0.5 m H_2_SO_4_	Highly under‐coordinated Ru (CN = 3) sites can coordinate with more OH groups and reduce the energy barrier	Ru‐exchanged MOF derivative annealing
2	Cr_0.6_Ru_0.4_O_2_^[^ ^79^ ^]^	Ru^4+^	178 mV @ 10 mA cm^−2^	10 000 cycles	0.5 m H_2_SO_4_	A lower energy barrier for the formation of *OOH (RDS)	Ru‐exchanged MOF derivative annealing
3	Mn doped RuO_2_^[^ ^113^ ^]^	Ru^4+/5+^	158 mV @ 10 mA cm^−2^	5000 cycles	0.5 m H_2_SO_4_	Decrease free energy of the rate‐determining step	Ru‐exchanged MOF derivative annealing
4	Co doped RuO_2_^[^ ^114^ ^]^	Ru^4+^	200 mV @ 10 mA cm^−2^	1000 cycles	0.5 m H_2_SO_4_	Balance adsorption free energy of intermediates by the modulation of d‐band center	A facile wet‐chemical method and a post‐annealing treatment
5	W_0.2_Er_0.1_Ru_0.7_O_2–*δ*_^[^ ^73^ ^]^	Ru^4+^	168 mV @ 10 mA cm^−2^	250 h @ 10 mA cm^−2^	0.5 m H_2_SO_4_	Decrease adsorption energies for oxygen intermediates	hydrothermal method with a post‐annealing
6	Cu_0.3_Ir_0.7_O_*δ*_^[^ ^74^ ^]^	Ir^4+^	351 mV @10 mA cm^−2^	6000 s @ 1.68 V	0.1 m HClO_4_	Balanced the free energy of each step for OER	Hydrothermal method with a post‐annealing
7	5% Gd doped IrO_2_^[^ ^115^ ^]^	Ir^3+/4+^	287 mV @10 mA cm^−2^	6 h @ 10 mA cm^−2^	0.5 m H_2_SO_4_	Facilitate the adsorption of water molecules and deprotonation	Template‐free ammonia complex‐based method
8	Mn doped IrO_2_^[^ ^116^ ^]^	Ir^3+/4+^	267 mV @ 10 mA cm^−2^	–	1 m KOH	Increase oxygen vacancy; enhance Ir–O interaction	Modified Wohler's method
9	Pt_0.1_La_0.1_‐IrO_2_@NC^[^ ^81^ ^]^	Ir^4+^	205 mV @ 10 mA cm^−2^	135 h @ 10 mA cm^−2^	0.5 m H_2_SO_4_	Tune d band centers; lower the energy barrier of RDS	Ir‐exchanged MOF derivative annealing
10	Ir_0.5_Ni_0.2_Co_0.3_O_*δ*_^[^ ^117^ ^]^	Ir^4+^	285 mV @10 mA cm^−2^	20 000 s @ 10 mA cm^−2^	0.1 m HClO_4_	Optimize surface–oxygen interaction energy; improve surface reaction kinetics	Hydrothermal method

### 7.1 Perovskite Oxides

**Figure 15:**
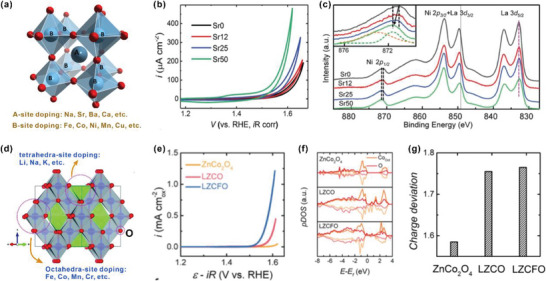
Design of high‐valence perovskite and spinel catalysts for OER. a) Schematic of high‐valence perovskite structure with A/B‐site doping. b) Cyclic voltammetry (CV) of LSNO films measured in O_2_ saturated 0.1 m KOH at a scan rate of 10 mV s^−1^, normalized by the specific area with voltage corrected for the electrolyte resistance. c) La 3d and Ni 2p XPS measured in situ for the LSNO film series. All spectra are shifted so the associated O 1s peaks fall at 530.0 eV. Inset: Ni 2p1/2 spectra for which the peak shift with *x* is clearly seen. Reproduced under the terms of the Creative Commons Attribution License.^[^ ^88^ ^]^ Copyright 2019, The Authors, published by Wiley‐VCH. d) Summary of the doping strategies in high‐valence spinel structure. Reproduced with permission.^[^ ^102^ ^]^ Copyright 2020, Royal Society of Chemistry. e) OER CVs, f) Computed pDOS, and g) Charge deviation of octahedral Co of ZnCo_2_O_4_, Li_0.5_Zn_0.5_Co_2_O_4_ (LZCO) and Li_0.5_Zn_0.5_Fe_0.125_Co_1.875_O_4_ (LZCFO). Reproduced with permission.^[^ ^64^ ^]^ Copyright 2019, Wiley‐VCH.

Perovskite oxides are characterized by a structural formula of ABO_3_. A is usually a rare‐earth or alkaline‐earth metal with a relatively large ionic radius, which is coordinated with 12 oxygen ions, whereas B is generally a transition metal (TM) with relatively small radius, such as 3d (Mn, Fe, Co, Ni, Cu, etc.), 4d (Mo, Nb, Pd, etc.), and 5d TMs (Hf, Ta, and W), forming a corner‐shared octahedral structure with oxygen ions.^[^ ^84^ ^]^ Versatilities of elemental composition and crystal structure for perovskite oxides provide vast space for modification of the electronic configuration to improve the OER performance.^[^ ^85^ ^]^ A‐site tuning, B‐site tuning, or A‐ and B‐site dual tuning are common strategies for synthesis of high‐valence perovskites (**Figure** **15**a), e.g., by solid‐state synthesis or sol–gel synthesis with annealing (Table 1).

#### 7.1.1 A‐Site Tuning

A‐site substitution by relatively low‐valence metals is an effective way of driving up the valence of the B‐site element to derive high‐valence perovskite oxides due to the total charge balance. The enhanced valence state for the B‐site element can strengthen the covalency of B‐site element and oxygen, enhancing the electronic configuration and conductivity to promote the OER activity. The common low‐valence metals for A‐site tuning of perovskite oxides are alkaline‐earth metals (like Ca, Sr, Ba, etc.)^[^ ^13^, ^86^, ^87^, ^88^ ^]^ or alkali metal (Li, Na, K, etc.).^[^ ^13^, ^86^, ^87^, ^88^, ^89^ ^]^ For instance, a La_0.5_Sr_0.5_NiO_3_ (Ni^3+/4+^) exhibits a reduced overpotential of 290 mV even at a relatively high current density of 50 µA cm^−2^, lower than that of 343 mV of LaNiO_3_^[^ ^88^ ^]^ (Figure 15b). The Ni oxidation state is raised by the Sr^2+^ substitution, Figure 15c, to enhance the Ni 3d–O 2p hybridization, then the electron extraction from oxygen adsorbates, and thus increase the OER activity. In a similar structure, the OER performance of LaCoO_3_ is improved by Sr^2+^ substitution in the La^3+^ site (Co^3+/4+^).^[^ ^19^ ^]^ The increasing oxygen vacancies and efficient oxygen diffusion rate induced by the Sr^2+^ substitution facilitate OER to proceed via LOM. Moreover, a Sr_0.9_Na_0.1_RuO_3_ (Ru^4+/5+^) catalyst also exhibits an extremely low potential of 1.4 V under 10 mA cm^−2^ and retains 85% activity after 20 cycles in an acidic electrolyte.^[^ ^89^ ^]^ In contrast, the undoped SrRuO_3_ catalyst is rather unstable in acidic solutions and even becomes inactive after the first cycle.

In addition to partial A‐site substitution to develop a mixed valence of perovskites, a completely high‐valence perovskites, based on Co^4+^, is also produced. Grimaud et al.^[^ ^8^ ^]^ have demonstrated that nonstoichiometric SrCoO_3−*δ*_ possesses superior OER activity than LaCoO_3_ and Sr_x_La_1−x_CoO_3_ due to the strong covalency of Co^4+^–O and the efficient LOM pathway involved. Then stoichiometric CaCoO_3_ without oxygen vacancies, was also developed under high pressure by Goodenough's group.^[^ ^10^ ^]^ The Co^4+^ ion in CaCoO_3_ can be stabilized under high pressure and the itinerant σ* antibonding endows it a metallic behavior. CaCoO_3_ exhibits a higher OER activity than LaCoO_3_ because of a shorter Co—O bond length (1.867 Å) and a larger σ* bandwidth.

#### 7.1.2 B‐Site Tuning

B‐site tuning is also an effective way of modifying the electronic structure and enhancing the valence of the central ion.^[^ ^90^ ^]^ Metal elements with lower valence state or higher electronegativity (like Fe, Co, Cu, etc.) are usually applied as B‐site dopants, and then the modified electron density of the B‐site can promote charge transfer with oxygen anions with increased B‐site metal and oxygen covalency.

For instance, Ede et al.^[^ ^91^ ^]^ report that Sr_2_Co_1.5_Fe_0.5_O_6–*δ*_ exhibits an improved OER activity due to partial substitution of Co by Fe. The overpotential of Sr_2_Co_1.5_Fe_0.5_O_6–*δ*_ catalyst was 318 mV at a current density of 10 mA cm^−2^, nearly 70 mV lower than that of the undoped counterpart. Moreover, partial substitution of the B‐site elements enhances the OER for LaBO_3_ (B = Co, Ni). For example, Xu's group^[^ ^92^ ^]^ synthesizes LaCo_0.9_Fe_0.1_O_3_ that exhibits a current density of 0.272 mA cm^−2^_oxide_ at an overpotential of 0.4 V, nearly twice that of the undoped LaCoO_3_. DFT simulations indicate that the superior activity of the half‐metallic LaCo_0.9_Fe_0.1_O_3_ is due to the increased spin state of Co^3+^ and enhanced covalency originated from the strengthened Co 3d and O 2p hybridization. Moreover, a LaNi_0.9_Cu_0.1_O_3_ (M = Cu, Co) nanosheet catalyst shows higher OER activity with lower onset potential and overpotential than the pure LaNiO_3_,^[^ ^93^ ^]^ the substitution of Ni^3+^ by Cu^2+^ strengthened lattice strains and oxygen vacancies, to promote the OER activity.

#### 7.1.3 A‐B‐Site Dual Tuning

Dual doping of both the A‐ and the B‐sites taps on the synergistical effect to optimize the electronic configuration of the parent catalyst, thus enhancing the electrocatalytic activity of the prepared OER catalyst.^[^ ^86^ ^]^ The concept has been successfully demonstrated, e.g., in Ba_0.5_Sr_0.5_Co_0.8_Fe_0.2_O_3–*δ*_ (BSCF) by dual doping of Sr and Fe elements,^[^ ^14^ ^]^ with a remarkable OER potential of 1.48 V at 50 µA cm^−2^_ox_. Moreover, its intrinsic activity is even one order of magnitude higher than that of the benchmark IrO_2_ catalyst. Kim et al.^[^ ^20^ ^]^ also show highly active La_0.2_Sr_0.8_Co_0.8_Fe_0.2_O_3–*δ*_ (LSCF) and Ba_0.5_Sr_0.5_Co_0.8_Fe_0.2_O_3–*δ*_ (BSCF) catalysts, with the concurrent incorporation of foreign metals in both A and B sites. Liu's group^[^ ^42^ ^]^ report a dual‐doping perovskite PrBa_0.5_Sr_0.5_Co_1.5_Fe_0.5_O_5+*δ*_ nanofiber with a mass activity 72 times higher than that of the undoped PrBaCo_2_O_5+*δ*_ and 2.5 times higher than that of the state‐of‐the‐art IrO_2_ at an overpotential of 0.37 V. The improvement is attributed to the synergetic effect of an optimized electronic configuration, efficient charge transfer and increased surface area. In another case, Porokhin and co‐workers^[^ ^94^ ^]^ identify that a La_0.6_Ca_0.4_Fe_0.7_Ni_0.3_O_2.9_ exhibits an improved mass activity of 400 A g^−2^_ox_ at 1.61 V, confirming that the increased valence states of Ni/Fe, oxygen vacancies and upshifted O p‐band center synergistically promote the activation of the lattice oxygen oxidation (LOM) mechanism to enhance its OER activity. Qu and co‐workers^[^ ^95^ ^]^ also demonstrate that a dual‐site doped La_1.4_Sr_0.6_NiMoO_6_ catalyst owns its excellent OER activity to its high‐valent Ni^3+^ states and upshifted O 2p center.

### 7.2 Spinel Oxides

The structure of spinel oxides is characterized by AB_2_O_4_, in which A and B are generally transition metals.^[^ ^85^ ^]^ Both octahedral (*oh*) and tetrahedral (th) structures coexist in the spinel structure. Spinel oxides may be divided into normal spinels, where A^2+^ ions stay at the *th* sites and B^3+^ ions stay at the *oh* sites, and inverse spinels, in which half of the B^3+^ ions occupy the *th* sites while A^2+^ and the rest of B^3+^ occupy the *oh* sites.^[^ ^96^ ^]^ The d‐orbital splitting of the metal ions located in the two sites are different, which influence the electronic configuration of spinel oxides and thus change their OER performance. High valent spinel oxides are generally prepared by (co‐)doping and the synthesis methods are analogous to those for perovskite oxides, such as solid‐state synthesis or liquid‐state synthesis accompanied by subsequent annealing (Figure 15d and Table 2).

#### 7.2.1 Normal Spinel Oxides

For normal spinel oxides, Co_3_O_4_ is a well‐known catalyst for OER, but its activity is still inferior to the benchmark of RuO_2_ or IrO_2_. Many modulate its crystal structure by doping, e.g., with Zn, Fe, Ni, Cu, V, Cr, Ag, and Li. Liu et al.^[^ ^97^ ^]^ report that Zn_0.75_Co_2.25_O_4_ pillar arrays show a low overpotential of 320 mV at 10 mA cm^−2^, better than that of the undoped Co_3_O_4_ and the commercial Ir/C. The replacement of Co ions with Zn^2+^ facilitates the formation of Co^4+^ species, which can accelerate the charge transfer between Co cations and O anions to promote the water oxidation process. In addition, by partial replacement of Zn with Li in the tetrahedral site, Li_0.5_Zn_0.5_Co_2_O_4_ and Li_0.5_Zn_0.5_Fe_0.125_Co_1.875_O_4_ catalysts exhibit superior OER activity, even higher than IrO_2_, due to a strong electronic overlap of octahedral Co and O. The partial substitution of Zn^2+^ with Li^+^ promotes charge transfer from oxygen to active Co_oh_ centers and strengthen the Co_oh_–O interaction^[^ ^64^ ^]^ (Figure 15e–g).

#### 7.2.2 Inverse Spinel Oxides

CoFe_2_O_4_ is a typical inverse spinel structure. In an early study, Ni or Mn is doped in the Fe site to prepare CoFe_2–x_Ni_x_O_4_ and CoFe_2–x_Mn_x_O_4_ catalysts.^[^ ^98^ ^]^ A CoFe_1.7_Ni_0.3_O_4_ displays the best OER performance with its overpotential reduced from 454 to 430 mV at a current density of 1 mA cm^−2^. The displacement of Fe^3+^ with Ni^2+^ changes the oxidation state of Co cations, enhancing the Co^2+^/Co^3+^ pair in the *oh* site for superior OER. Recently, Chen and co‐workers^[^ ^99^ ^]^ synthesized an inverse LiCoVO_4_ spinel oxide. The transition from a normal to an inverse spinel oxide occurs by incorporation of Li^+^ and V^5+^ into ZnCo_2_O_4_, where Co^2+^ stays in the *oh* site, whereas Co^3+^ in the *th* site. The OER activity shows a sharp increase from that of ZnCo_2_O_4_ and the overpotential reach 290 mV@1 mA cm^−2^. The experimental and calculation results demonstrate that the Co^2+^ in LiCoVO_4_ is stabilized at the active *oh* sites at high spin states *S* = 3/2 (*t*_2g_^5^ *e*_g_^2^) and the incorporation of Li and V also increase the Co–O covalency.

### 7.3 Pyrochlore Oxides

**Figure 16:**
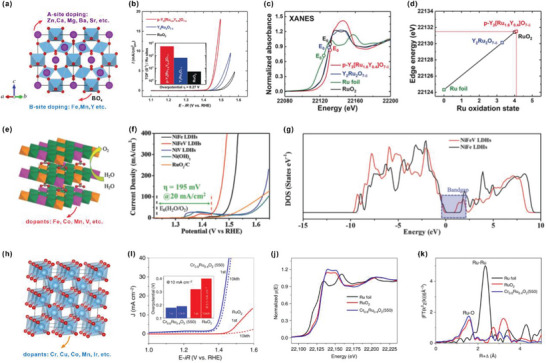
a) High‐valence pyrochlore (A_2_B_2_O_7_) structures synthesized by different site doping. Reproduced under the terms of the Creative Commons Attribution License.^[^ ^118^ ^]^ Copyright 2019, The Authors, published by the Royal Society. b) CVs and corresponding TOFs (inset) of porous Y_2_[Ru_1.6_Y_0.4_]O_7−*δ*_, Y_2_Ru_2_O_7−*δ*_ and RuO_2_ electrocatalysts. c) Normalized Ru K‐edge XANES spectra with absorption energy (*E*_0_) of porous Y_2_[Ru_1.6_Y_0.4_]O_7−*δ*_ and Y_2_Ru_2_O_7−*δ*_ with Ru foil and RuO_2_ as references. d) Ru oxidation state as a function of *E*_0_. Reproduced with permission.^[^ ^107^ ^]^ Copyright 2018, Wiley‐VCH. e) The schematic diagram of high‐valence LDHs structure via different metal doping. f) Polarization curves of the non‐noble metal catalysts (NiFeV LDHs, NiFe LDHs, Ni(OH)_2_, and NiV LDHs) and the commercial RuO_2_/C catalyst. g) Total density of states (TDOS) curves of NiFeV LDHs and NiFe LDHs, the narrower bandgap of NiFeV LDHs indicates a more conductive structure. Reproduced with permission.^[^ ^69^ ^]^ Copyright 2018, Wiley‐VCH. h) High‐valence rutile structure synthesized by doping other metal ions. Reproduced with permission.^[^ ^119^ ^]^ Copyright 2014, Elsevier. i) LSVs of Cr_0.6_Ru_0.4_O_2_ (550) and commercial RuO_2_ for the first and 10 000th cycle. Inset shows the comparison of overpotentials for Cr_0.6_Ru_0.4_O_2_ (550) and RuO_2_ at the current density of 10 mA cm^−2^. For RuO_2_ after 10 000 cycles, the overpotential is corresponded to 3.5 mA cm^−2^. j) Normalized Ru K‐edge XANES spectra and k) Fourier transformed EXAFS spectra of Cr_0.6_Ru_0.4_O_2_ (550), Ru foil and commercial RuO_2_. Reproduced under the terms of the Creative Commons Attribution 4.0 International License.^[^ ^79^ ^]^ Copyright 2019, The Authors, published by Springer Nature.

Another type of TM oxides is pyrochlore oxides, which could be expressed in the formula of A_2_B_2_O_7_, where A is usually one of the rare‐earth or alkaline‐earth metal elements and B is generally a transition metal element. Among those, ruthenium based pyrochlore oxides are recognized as promising OER catalysts due to the strong covalency of the Ru—O bond. Some studies have confirmed that improving the valence of Ru in pyrochlore oxides can promote their OER catalytic activity.^[^ ^11^, ^103^ ^]^ Therefore, designing high‐valence ruthenium based pyrochlore oxides is of significance for water oxidation reactions. Low‐valence doping in the A site or the B site, e.g., by Zn, Ca, Mg, Ba, Co, and Cu, is an effective strategy to prepare high‐valence Ru‐based pyrochlore structures. Similarly, the incorporation of the foreign elements modify the electronic configuration of the pristine materials and improve the covalency of B‐site metal with O and the electrical conductivity, thus improving the OER activity of the catalysts (**Figure** **16**a). The preparation method of high‐valence pyrochlore oxides includes solid‐state preparation, sol–gel method with a post‐calcination, or hydrothermal method (Table 3).

#### 7.3.1 A‐Site Tuning

A‐site tuning by doping is a usual approach in preparing high‐valent pyrochlore oxides. Wang's group^[^ ^103^ ^]^ systematically investigates the A‐site doping of Y_2_Ru_2_O_7_ catalysts by a series of low valent elements, Zn, Ba, Mg, Ca, etc. For example, a highly active and stable Y_1.85_Zn_0.15_Ru_2_O_7–*δ*_ OER catalyst shows a remarkable overpotential of 290 mV at 10 mA cm^−2^, lower than that of the undoped structure and remained stable in an acidic electrolyte under the current density of 10 mA cm^−2^. The partial substitution of Y^3+^ by Zn^2+^ contributes to the increased valency of Ru from Ru^4+^ to a mixed Ru^4+^ and Ru^5+^, as well as promoted level of oxygen vacancies, to accelerate OER reaction kinetics and activity. Subsequently, Y_1.85_Ba_0.15_Ru_2_O_7–*δ*_,^[^ ^104^ ^]^ Y_1.85_Mg_0.15_Ru_2_O_7_,^[^ ^105^ ^]^ and Y_1.75_Ca_0.25_Ru_2_O_7_^[^ ^106^ ^]^ catalysts are developed with comparable OER activity and stability. Moreover, Zhang et al. also demonstrate that A‐site substitution of Sr^2+^ for Y^3+^ can optimize the electronic states of Y_2_Ru_2_O_7_ and further improves the intrinsic OER performance in an acidic solution.^[^ ^65^ ^]^

Apart from the mixed valence Ru^4+/5+^ in the pyrochlore ruthenate, there are also attempts to prepare complete high‐valence pyrochlore oxides with Ru^5+^. For instance, Shigeto et al.^[^ ^11^ ^]^ achieve a non‐Fermi liquid Hg_2_Ru_2_O_7_ catalyst with only Ru^5+^ under 950 °C and 6 GPa, which shows much better OER activity than RuO_2_, with an ultra‐low overpotential of 150 mV at 10 mA cm^−2^. The coexistence of the localized d‐bands and the metallic state in the non‐Fermi liquid is the key factor for the remarkable OER performance.

#### 7.3.2 B‐Site Tuning

B‐site tuning by doping is another strategy to prepare high valent pyrochlore oxides. Yang's group^[^ ^107^ ^]^ successfully obtain a Y_2_Ru_1.6_Y_0.4_O_7–*δ*_ with the substitution of Y^3+^ into Ru^4+^ sites. The current density of Y_2_Ru_1.6_Y_0.4_O_7–*δ*_ at 1.5 V can be up to 18.1 mA cm^−2^ in a HClO_4_ solution, nearly twice that of the undoped Y_2_Ru_2_O_7–*δ*_ (9.49 mA cm^−2^) (Figure 16b). The mixed oxidation state of Ru^4+/5+^ and increased oxygen lattice defects greatly contribute to the excellent OER activity (Figure 16c,d). Recently, Han and co‐workers^[^ ^108^ ^]^ synthesize Y_2_Ru_1.9_Mn_0.1_O_7–*δ*_ and Y_2_Ru_1.9_Fe_0.1_O_7–*δ*_ as OER catalyst. Compared with Y_2_Ru_2_O_7_, Y_2_Ru_1.9_Mn_0.1_O_7–*δ*_ displays improved activity but inferior stability, while Y_2_Ru_1.9_Fe_0.1_O_7–*δ*_ shows little change in activity but significant enhancement in stability. XPS results revealed that Mn, Fe substitution of the B‐site in Y_2_Ru_2_O_7_ leads to a mixed Ru^4+/5+^ valence and considerable oxygen vacancies in Y_2_Ru_1.9_Mn_0.1_O_7–*δ*_ and Y_2_Ru_1.9_Fe_0.1_O_7–*δ*_. DFT calculations illustrate that Fe ions stabilizes the structure by increase of the surface dissolution energy and the Ru–O covalency, which prevents the Ru atom dissolution in an acidic solution.

### 7.4 Layered Double Hydroxide (LDHs)

TM hydroxides contains hydroxides, oxyhydroxides, and layered double hydroxides (LDHs). Among those, FeNi LDHs and FeCo LDHs are two representative catalysts for OER. Incorporation of foreign elements (e.g., Cr, V, Zr, Zn) into the lattice of LDHs is a typical method of preparing high‐valence FeNi LDHs or FeCo LDHs^[^ ^109^, ^110^ ^]^ (Figure 16e). There are many ways of introducing foreign atoms into FeNi LDHs or FeCo LDHs, such as hydrothermal, co‐precipitation, electrodeposition as well as etching and dissolution (Table 4).

#### 7.4.1 CoFe LDHs

A gelled FeCoW oxyhydroxides, synthesized from the sol–gel method with post‐refluxing, shows a low overpotential of 191 mV at 10 mA cm^−2^ and exhibits excellent stability even after 500 h stability test.^[^ ^111^ ^]^ The Co ions in the G‐FeCoW oxyhydroxides is readily oxidized to a relatively high valence due to the existence of W^6+^, explaining its excellent OER activity. The common method of preparing high‐valence CoFe LDHs is hydrothermal and co‐precipitation. Moreover, a hydrothermally synthesized Cr‐doped CoFe LDH on nickel form (NF), CoFeCr LDH/NF, achieves a small overpotential of 202 mV at 10 mA cm^−2^, and an outstanding durability without any current decline for 20 h.^[^ ^71^ ^]^ The strong electrophilic Cr^6+^, which is spontaneously oxidized during the OER, draws electrons from the Co sites to stabilize the active sites to a high oxidation state. The high‐valence Co reduced the energy barrier of H_2_O adsorption and accelerated the OER.

#### 7.4.2 NiFe LDHs

Li et al.^[^ ^69^ ^]^ prepared NiFeV LDHs as OER catalyst through hydrothermal and co‐precipitation method. NiFeV LDHs possessed an extremely low overpotential of 195 at the current density of 20 mA cm^−2^ and excellent stability with 98% maintained after 18 h at the potential of 1.48 V (Figure 16f). The experimental and simulation results reveal that the *V* doping tunes the electronic configuration NiFe LDHs by narrowing the gap between the valence and the conduction bands and improves its electrical conductivity, thereby boosting efficient electron transfer and favoring its OER performance (Figure 16g). In addition, a Ni_3_FeAl_0.91_ LDHs/NF catalyst, from partial etching/dissolution of Al^3+^ in strong alkaline solution, yields a current density of 20 mA cm^−2^ at a low overpotential 304 mV.^[^ ^70^ ^]^ The enhanced OER activity is mainly attributed to an increased level of Ni^3+^ induced by theAl^3+^ substitution. Moreover, partial Al^3+^ dissolution creates more defects and further facilitates the exposure of Ni active sites on the surface.

### 7.5 Rutile Oxides

The representative rutile oxides for OER are IrO_2_ and RuO_2_, both of which are regarded as the benchmark to evaluate alternative OER catalysts due to their intrinsically outstanding OER activity.^[^ ^86^, ^112^ ^]^ High‐valence RuO_2_‐based and IrO_2_‐based oxide catalysts are also prepared by low‐valence metal doping, such as Mg, Cr, Mn, Cu, Zn, Co, and W, to promote the electronic configurations of the pristine catalysts and thus their OER performance (Figure 16h). Moreover, many studies illustrate that RuO_2_/IrO_2_ based catalysts suffer from poor stability at high anodic voltages or under acid electrolytes, since RuO_2_ or IrO_2_ will transform to soluble RuO_4_ or IrO_3_, respectively.^[^ ^112^ ^]^ Thus, the incorporation of foreign metal ions should also aim to stabilize Ru or Ir in the lattice (Table 5).

#### 7.5.1 RuO_2_‐Based Structures

A variety of synthesis strategies are reported to prepare high‐valence Ru‐based oxides. For instance, Chen's group has successfully doped Cu into the porous polyhedral interstices of RuO_2_^[^ ^80^ ^]^ and Cr_0.6_Ru_0.4_O_2_^[^ ^79^ ^]^ by the pyrolysis of MOFs, with the precursor of Cu‐BTC (HKUST‐1) and MIL‐101 (Cr), respectively. Both of the resulting compounds exhibit amazing OER activity with an overpotential of ≈180 mV at the current density of 10 mA cm^−2^ in a 0.5 m H_2_SO_4_ solution and an excellent stability even after 10 000 cycles (Figure 16i). Experimental results and DFT calculations confirm that the Cu/Cr doping optimizes the electronic configuration and increased the covalency of Ru–O, to enhance the OER activity and prevent the formation of soluble RuO_4_ on the surface of the catalysts (Figure 16j,k). Similarly, Chen and co‐workers^[^ ^113^ ^]^ developed Mn‐doped RuO_2_ using Mn‐BTC as the precursor, achieving admirable OER acidic activity and stability with an overpotential of 158 mV at 10 mA cm^−2^ and negligible current density loss after 5000 cycles. The incorporation of Mn regulates Ru d‐band center and weakens the antibonding surface‐adsorbate states, thus reducing the energy of the rate‐determining step and increasing the intrinsic activity.

Apart from the pyrolysis of MOFs, ultrathin M (M = Fe, Co, or Ni) doped RuO_2_ networked nanowires (NWs) are also achieved through wet‐chemical reduction and post‐calcination,^[^ ^114^ ^]^ along with abundant structural defects and grain boundaries, to improve the OER performance. The transition metal doping modulates Ru d‐band center and then balance the adsorption energy for oxygen intermediates, leading to the enhancing OER activities. Among these catalysts, Co‐doped RuO_2_ shows the lowest overpotential of 200 mV when reaching the current density of 10 mA cm^−2^.

### 7.6 IrO_2_‐Based Structure

Similar to RuO_2_, the activity and stability of IrO_2_ are improved by modulation of the elemental chemistry, valence states, and electronic configuration. For instance, a Cu doped IrO_2_,^[^ ^74^ ^]^ Cu_0.3_Ir_0.7_O_*δ*_, exhibits a low overpotential of 351 mV in an acidic solution with an excellent stability. The incorporated CuO_6_ octahedron leads to a stronger Jahn–Teller distortion and induces abundant oxygen defects. The distorted IrO_6_ octahedral structure made the d_z_^2^ orbital partially occupied, which balances the free energies of each OER step and ultimately reduces the overpotential. In another report, Wang and co‐workers^[^ ^115^ ^]^ show a promising catalyst of Gd‐doped porous IrO_2_ with the overpotential of 287 mV @10 mA cm^−2^ by a template‐free ammonia‐complex based method. Experimental and theoretical results reveal that the substitution with Gd^3+^ increases the ratio of Ir^4+^/Ir^3+^ to accelerate H dissociation, and the Gd^3+^ doping also stimulates the formation of oxygen vacancies to promote the adsorption of H_2_O molecules. Lee and co‐workers^[^ ^116^ ^]^ synthesize a series of M (M = Cr, Mn, Fe, Co, Ni) doped IrO_2_ nanoparticles (NPs) by a modified Wohler's method. Among those, Mn doped IrO_2_ yields the lowest overpotential of 267 mV at 10 mA cm^−2^, nearly 50 mV lower than that of pure IrO_2_. The extraordinary catalytic activity of the Mn‐doped IrO_2_ originates from the high level of the oxygen vacancy defects, which is negligible for Ni doped IrO_2_.

Dual‐site doped IrO_2_ catalysts also exhibit superior water oxidation activities. For instance, a Pt_0.1_La_0.1_‐IrO_2_@NC, synthesized from a MOF template (ZIF‐8), offers remarkable OER performance with an overpotential of 205 mV@10 mA cm^−2^ and excellent stability for 135 h under an acidic solution.^[^ ^81^ ^]^ DFT results confirm that the Pt and La co‐doping into IrO_2_ modulates the Ir d‐band center to reduce the energy barrier from *O and *OOH (RDS). In another case, Zaman et al.^[^ ^117^ ^]^ show a Ni‐Co co‐doped IrO_2_, Ir_0.5_Ni_0.2_Co_0.3_O_*δ*_ from hydrothermal synthesis, gives rise to the lowest overpotential of 285 mV @10 mA cm^−2^ among the single‐site doped and undoped IrO_2_. The Ni/Co co‐doping broadens the Ir‐5d band and enlarges the overlap of the Ir 5d‐O 2p, thus accelerating surface reaction kinetics.

## 8 Conclusions and Perspectives

Oxygen evolution reaction (OER) is a critical half reaction in metal‐air batteries, water splitting hydrogen generation, and other oxygen‐involved electrochemical up‐conversion processes. Fundamental understanding of the mechanism of OER and rational design of highly active and cost‐efficient electrocatalysts are of great importance, to develop high energy density rechargeable batteries or to generate green hydrogen and other value‐added chemicals. Very recently, engineering the valency of transition‐metal oxides has been shown to be an effective approach to achieve far higher OER performance than their low‐valence counterparts.

Here we have firstly summarized the critical roles of HVOs as OER catalysts based on two completing mechanistic pathways: AEM and LOM. For AEM, high‐valence states optimize the electronic configuration of the *e*_g_ orbital filling, which balances the binding energy between the catalytic sites and the oxygen intermediates. Moreover, the strong covalency of M–O in HVOs can promote the charge transfer between surface metal cations and adsorbates, therefore accelerating the OER. Furthermore, the elevated oxidation state of TMOs leads to a small band gap and better electrical conductivity, which effectively reduces the Ohmic potential drop and energy loss between the catalyst and the current collector. Moreover, HVOs are energetically favorable for the more efficient LOM pathway because the enhanced valence downshifts the metal d band to below the O p band, which activate the lattice oxygen as the redox center to donate electrons to the external circuit directly. Moreover, the large concentration of oxygen vacancies and fast deprotonation of the high‐valence effect are also favorable for the LOM. In addition, we also summarize the synthesis strategies and the recent advances of HVOs as OER catalysts. Most of the reported HVOs employ the strategy of elemental doping during the synthesis, including high‐temperature solid‐state, sol–gel, solvothermal, and pyrolysis approaches. The other two effective synthesis strategies are high‐pressure synthesis in diamond anvils, and de‐lithiation/de‐sodiation from layered oxides, such as LiCoO_2_ and NaNi_y_Fe_1−y_O_2_. The advantages and disadvantages of each are critically compared and discussed. Furthermore, the recent advances of HVOs are summarized in categories of crystal structures, including perovskite, spinel, pyrochlore, TM hydroxide, and noble Rutile oxide (Ru/Ir oxides).

Although significant progress has been made in the design and in‐depth understanding of HVOs for OER catalysis, the commercial utilization of those remains challenging. Future efforts should be made to overcome the following issues.

1) How to reduce the particle size of high‐valence oxides: As mentioned above, a large number of high‐valence oxides are prepared at high temperature and/or even high pressure, which usually lead to relatively large particle sizes, typically from 0.1 to 2 µm, which is not atomically efficient for a catalyst for practical applications. Catalysts with a large specific surface area possess more active sites and generate high OER current density. To exclude the geometric effects, many researchers propose to compare the intrinsic OER activities normalized by BET or ECSA, which is important for exploration of the intrinsic mechanisms, the real active sites and the RDS. However, for full‐cell design, the actual activity is the most important parameter. Therefore, how to reduce the particle size of the structures is the next challenge for the applications of HVOs. It is necessary to develop strategies to enhance the nucleation and prevent the growth or agglomeration of large particles during synthesis, as discussed, e.g., in a recent topical review.^[^ ^120^ ^]^ Alternative approaches to controlling the grain sizes may include low‐temperature post‐treatment, porous‐electrode templating^[^ ^121^ ^]^or mechanochemical synthesis.^[^ ^122^ ^]^
2) Developing mild but high‐yield synthesis: Non‐noble TMOs are desirable as alternatives for precious metal Ru‐/Ir‐based oxides due to low cost of chemical resource. However, due to the relatively large thermodynamic formation barrier, HVOs are usually obtained under rigorous conditions, including long‐term high temperature annealing, high pressure, or high applied voltage. The rate of yield is rather low, even several milligrams every time from the diamond anvils, which is impractical for commercial applications. Electrochemical delithiation suffers from a purification challenge from the complex cathode materials, including conductive materials and binding agent. All of those greatly increase the cost of high‐valence catalysts, even more expensive than the noble metal oxides. Thus, attention should be paid to reducing the overall cost of catalysts, including resource, synthesis, and scalability. A simple, high‐yield and cost‐efficient method is desirable for highly active OER catalysts.
3) Developing bifunctional OER/HER or OER/ORR catalysts: At the full‐cell level, water splitting and metal‐air batteries involve two half‐reactions of OER/HER and OER/ORR, respectively. For the time being, Pt is the benchmark electrocatalysts for HER and ORR. However, two different catalysts in one integrated device, either an electrolyzer or a rechargeable air battery, not only creates manufacturing and integration complexity/cost, but also huge challenges in maximizing the performance due to the mismatched working conditions. As such, bifunctional catalysts are highly desirable for both full‐cell devices. Unfortunately, most of HVOs are unstable or inactive at the cathodic potential for ORR/HER (*U* < 1.23 V vs RHE for ORR and *U* < 0 V vs RHE for HER), although some bifunctional activities of HVOs are recently reported, such as Ir/Ni(OH)_2_ (HER/OER: Ir^5+^)^[^ ^123^ ^]^ and Mg‐doped LaNiO_3_ nanofibers (ORR/OER: Ni^3+/4+^).^[^ ^124^ ^]^ Specific structures with multi‐phases, such as core–shell structures and coordinated hybrids or complexes,^[^ ^125^, ^126^ ^]^ may be developed to stabilize the high‐valence metal sites at the cathodic potential.

In summary, considerable progress has been made in the fundamental understanding of the intrinsic mechanisms of HVOs as OER catalysts and several ingenious synthesis strategies for HVOs has also been reported in the past decade. Though challenges remain for commercialization, there is great scope for further electronic structural coordination and microstructural engineering to achieve large‐scale production of high‐valence oxide catalysts and the corresponding cost‐effective energy storage and conversion devices.

## Conflict of Interest

The authors declare no conflict of interest.
